# Local indigenous knowledge about some medicinal plants in and around Kakamega forest in western Kenya

**DOI:** 10.12688/f1000research.1-40.v2

**Published:** 2012-12-13

**Authors:** Nickson Erick Otieno, Caleb Analo

**Affiliations:** 1 National Museums of Kenya, Nairobi, Kenya; 2 Kakamega Environmental Education Project, Shinyalu, Kenya

## Abstract

Kakamega forest is Kenya’s only rainforest and is distinguishably rich in biodiversity but threatened by agricultural encroachment and other forms of human activity. It is also one of Kenya’s Important Bird Areas and a significant source of natural products to neighboring rural communities, such as medicinal plants, food, wood and other fibers. By using structured questionnaires for direct interviews, local indigenous knowledge was tapped through involvement of a focal group of elderly key informants in three blocks of the forest. Forty key species of medicinal plants used by local people were identified and recorded. Fifty-five percent of these were shrubs, thirty-two percent trees, seven-and-a-half percent lower plants such as herbs or forbs while five percent were climbers. About seventy percent of the medicinal plants occurred inside the forest itself and thirty percent around the edge and the immediate surroundings outside the forest. Thirty-eight (95%) of the plants were indigenous to Kenya and two (5%) exotic. Such extensive indigenous knowledge of the medicinal uses of the plants, including their distribution trends in the forest, may be tapped for decision support in rural health service planning, policy formulation for conserving the forest, tracking and mitigation of climate change impacts.

## Introduction

Although community development goals are not always consistent with biodiversity conservation objectives ^1^ there are often many opportunities for mitigating negative effects by tapping into local indigenous knowledge with reference to certain aspects of environmental use and conservation ^2^. Indeed, application of knowledge and values of communities that are resident within or around key biodiversity areas has been gaining increasing global popularity as significant elements in enriching and improving strategies for conserving biodiversity ^2^. This is because integration of such indigenous knowledge into conservation programs facilitates cross-borrowing of ideas, promotes constructive engagement, and instills a sense of common ownership and responsibility towards achievement of a synergy of goals ^3^. This echoes the concept of social capital ^3^ that, apart from amassing local support and goodwill, adoption of local indigenous knowledge in conservation may also promote and provide sustainable insurance against conflicts of purposes. This results in increased chances of achieving the dual goal of biodiversity conservation stewardship as well as community development. For instance, studies have shown that rainforest ethno-botanical checklists prepared by communities living in or near them tend to be more exhaustive because they are based on practical day-to-day uses that are firmly ingrained in local cultural norms and values ^3– 5^.

Like in many parts of the developing world, there is a growing upsurge in demand for herbal and other traditional remedies for various ailments among communities in Kenya. This is due either to the increasing cost of conventional modern medicine or, inadequacies in public health service delivery ^6^. For a long time, the bulk of “technical” information on traditional plant uses in the treatment of disease has been disparate and privately held, with limited accessibility to the public or peer-review domain ^7, 8^. Fortunately, over the past five years there has been an upsurge in research and publication on indigenous knowledge and use of medicinal plants in Kenya. This includes research on medicinal plants of the Nandi forest ^9^, indigenous knowledge on medicinal plants of Mt. Elgon forest ^10^ and the uses of medicinal plants by the Ogiek people of the East Mau forest ^11^. As a result, a firmer foundation is being laid gradually but steadily for further research into the effectiveness of these treatments and the various options for preparation and administration for managing diseases.

This study sought to set in motion a process for systematic documentation of plants of medicinal value in the Kakamega forest, with a view to consolidate indigenous knowledge about them and making this information available to the wider community. It is hoped that in the process of this, ecosystem and other socio-economic services offered by the Kakamega forest will be highlighted. The study also sought to highlight any plant species in the forest that may have medicinal value that are also of conservation concern, either as endangered or as invasive species.

## Materials and methods

**Figure 1.**
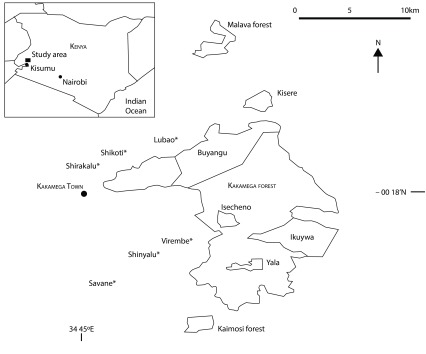
Map of Kakamega forest (this figure has been reproduced with kind permission from Otieno and co-authors ^13^).

**Study area:** The Kakamega forest lies in western Kenya between 00°08′30.5′′ – 00°23′12.5′′ N and 34°18′ 08′′ – 34°57′26.5′′ E from 1520–1680 m above sea level ^12– 14^ ( Figure 1). The mean annual rainfall is 2000 mm, with long rains in April/May and short rains in September/October ^12, 13^, while the mean annual temperature is 20°C. The forest covers 183 km ^2^ and 100 km ^2^ of this consists of closed canopy forest of which one-third, in the north, is gazetted as a national reserve under protection. The rest is comprised of grassy and bushed glades, tea, cultivation and plantations of softwoods and commercially valuable hardwoods ^14, 15^.

The forest is Kenya’s only true tropical rainforest ^14^ and constitutes one of Kenya’s 61 Important Bird Areas (IBAs) due to the presence of about 350 bird species, many of which are range-restricted or endemic species reminiscent of the wider Guineo-Congolean forest system that extended from the eastern Democratic Republic of Congo, of which Kakamega is the easternmost outlying relict ^15^. There are at least 380 plant species, though there is no significant endemism. As a result of massive exploitation through massive legal and illegal logging between the 1960s and 1990s, the forest flora is dominated by a mixture of large secondary-growth trees and hardly any primary-growth trees. Even for this secondary forest, much of the closed canopy and contiguity exists only in the northern part of the forest, consisting of the Buyangu blocks, which are now protected as a national wildlife reserve. The southern end, comprising of the Isecheno, Yala and Ikuywa as well as the detached units of the Kisere, Kaimosi and Malava blocks ( Figure 1), are managed as forest reserves but are still accessible to the local public community despite some level of official restriction.

Apart from birds and plants, the forest also has a remarkable richness in other biodiversity including several species of mammals, reptiles, amphibians and invertebrates ^16^.

The forest is under an increasing threat of loss to agriculture and settlement by the increasing local human population. The neighbourhood of the forest, where the western Kenya Bantu ethnic community called the Luhya reside, is densely populated with an average density of 433 persons per square kilometer ^17, 18^.

The study was carried out within the three main blocks of the southern Isecheno-Yala-Ikuywa blocks, Buyangu, and the detached Kisere and Kaimosi blocks in the north (see Figure 1). The blocks were covered in two field seasons of 11 days each, the first between April and May 2009 while the effects of the wet season were still evident and many plants bore fruit and then in late July during the dry season when full fruiting is reduced and some leaves are shed off. This was to control for any rainy-season effects.

**Sampling strategy:** A key informant was identified from each study block during each sampling week, to be interviewed about the medicinal plants as outlined by Kothari ^19^. The choice of blocks was primarily to achieve sampling with as much coverage of the forest as possible (including protected areas, reserves and detached fragments) though not necessarily to sample in every spatial part of the forest. Key informants were selected on the following criteria: (1) seniority of age in the community (not less that 50 years old); (2) local residency for a period of not less than 20 years; (3) knowledge of forest plants in the local dialect and well versed with their use(s). Current or previous experience as herbalist was preferable but not essential.

The selection was based on prior consultation with the local community leaders and additional guidance by field assistants according to Okello *et al.* ^10^ and prior consent was obtained before interviews. One of the key informants engaged in the interviews was a practicing traditional healer. Further information was obtained from random opportunistic interviews with 6 other non-core informants who were also at least 50 years old, 2 from each block. The choice of elderly informants was made on the basis that most indigenous knowledge about traditional medicine in developing countries tends to be possessed by elderly members of the society ^20^.

Data was collected from key informants through field excursions using direct personal interviews that employed the use of a structured questionnaire guided by a mix of closed and open-ended questions (see survey questionnaire). This was combined with free-style discussions and field excursions with the informants. For data consistency, the same informants were involved in each sampling season in each area. In addition, there was a final joint focused group discussion with all the key informants to synergize the information gathered. Information captured and recorded included:

1) Local name of plant in question; 2) Disease/condition treated by plant; 3) Plant part(s) used for the treatment; 4) Preparation method; 5) Indidgenous, common (English) and scientific name of the plants. These were determined by consulting illustrated standard botanical field guides ^7^.

Questionnaire provided to local informants to identify local medicinal plants The questionnaire provided to local informants to identify local medicinal plants

## Data analysis

A checklist of all recorded species of medicinal value was compiled, including their indigenous, common and scientific names, plant origination (i.e. indigenous or exotic), plant form (e.g. tree, shrub, herb etc.) and conditions treated ( Table 1 and data set). Data was also presented in terms of the methods of preparation and administration to patients; as well as the age group and gender of target patients (data file below). All the lists generated by the different key informants were scrutinized and synchronized into a final list at the joint focused group discussion ^20^. With help from the informants/respondents, each plant was observed in its natural habitat and a image taken using a digital camera, collected and pressed. For each medicinal plant, a small part (preferably one with flowers) was collected while fresh and digitally photographed for identification and pressed for herbarium. Species whose common (English) and scientific names were not immediately established in the field were taken for specialized identification at the EA Herbarium at the National Museums of Kenya in Nairobi.

**Table 1.** Checklist of the medicinal plants identified in and around Kakamega forest species accounts.

Scientific name	Local name	Common name	Family	Plant origin	Plant form	Diseases or conditions targeted
*Albizia grandi bracteata*	Mukhunzuli	Large-leaved Albizia	Fabaceae	Indigenous	Tree	Gonorrhea
*Albizia gummifera*	Musenzeli	Peacock flower	Fabaceae	Indigenous	Shrub	Sexually transmitted infections Stomach-ache
*Azadirachta indica*	*Muarubaini*	Neem tree	Meliaceae	Exotic	Tree	Fever, aches, pains Malaria attack Insect bites Pest control Skin infections
*Aspilia pluriseta*	Shralambila	Dwarf Aspilia	Asteraceae	Indigenous	Herb/forbe	Stopping bleeding in wounds Drippy nose in poultry
*Bequartiodendron oblanceolata*	Musamia	Not established	Not established	Indigenous	Tree	Ulcers in digestive track Boils around belly
*Chrysocephalum sp*	Mwikalo	Yellow Buttons	Asteraceae	Indigenous	Shrub	Stomach problems related to STIs
*Clematopsis scabiosifolia*	Lunyili	Not established	Ranunculaceae	Indigenous	Climber	Stuffy nose and associated respiratory problems
*Clerodendron pygmaeum*	Luseshe	Cashmere Bouquet	Verbenaceae	Indigenous	Shrub	Common flu and associated
*Coffea eugenioides*	Itikwa	Mufindi coffee	Rubiaceae	Indigenous	Shrub	Eye problems in livestock
*Conyza floribunda*	Liposhe	Asthma weed	Asteraceae	Indigenous	Shrub	Tooth-ache
*Desmodium adscendens*	Matite	Not established	Fabaceae	Indigenous	Herb/forbe	Stomach-ache
*Desmodium repandum*	Not established	Not established	Fabaceae	Indigenous	Shrub	Stomach upset
*Diospyros abyssinica*	Lusui	Giant Ebony	Ebenaceae	Indigenous	Tree	Recurrent nightmares Sores
*Dissotis speciosa*	Lunyili	Not established	Melastomataceae	Indigenous	Shrub	Diarrhea
*Dovyalis macrocalyx*	Shinavatevia	Shaggy-fruited dovyalis	Flacourtiaceae	Indigenous	Shrub	Constipation Peptic ulcers
*Entada abyssinica*	Shivayamboga	Abyssinia Entada	Leguminoceae	Indigenous	Tree	Stomach-ache
*Erythrococca atrovirens*	Shirietso	Not established	Euphorbiaceae	Indigenous	Shrub	Wounds, especially septic
*Hibiscus sp*	Lubulwa	Not established	Malvoideae	Indigenous	Shrub	Stomach-ache General fever
*Justica flava*	Lihululwa	Yellow Justicea	Acanthaceae	Indigenous	Herb/forbe	Reducing post-natal pains
*Lantana trifolia*	Imbulimutacha	Three-leaf Shrub	Verbenaceae	Indigenous	Shrub	Malaria and general fever (humans) Diarrhea in livestock
*Leucas calostachys*	Lumetsani	Not established	Lamiaceae	Indigenous	Shrub	Severe diarrhea especially accompanied with blood
*Leucas deflexa*	Shitsunzune	Not established	Lamiaceae	Indigenous	Shrub	Eye infection/effects in livestock
*Markhamia lutea*	Lusiola	Nile Tulip tree	Bignoniaceae	Indigenous	Tree	Ear pain in humans Eye problems in cattle
*Mondia whytei*	Mukomer	White’s ginger	Apocynaceae	Indigenous	Climber	Loss of appetite Low libido Fatigue Mineral deficiency
*Ocimum kilimandscharicum*	Not established	Kilimanjaro basil	Lamiaceae	Indigenous	Shrub	Nasal congestion, colds, flu, Insect bites General aches and pains
*Olea capensis*	Mutukhuyu	Elgon Olive	Oleaceae	Indigenous	Tree	Stomach-ache Peptic ulcers
*Paullinia pinnata*	Not established	Bread and cheese plant	Sapindaceae	Indigenous	Shrub	Hiccup
*Paulownia tomemtosa*	Musembe	Foxglove tree	Paulowniaceae	Exotic	Tree	Stomach problems Boils
*Piper capense*	Not established	Staart Pepper	Piperaceae	Indigenous	Shrub	Cough
*Piper umbellatum*	Indava	Cow-foot leaf	Piperaceae	Indigenous	Shrub	Head-ache and fever
*Plectrantus forsteri*	Shikhokho	Spur flower	Lamiaceae	Indigenous	Shrub	Worm infection in livestock
*Prunus africana*	Mwiritsa	Red Stinkwood	Rosaceae	Indigenous	Tree	Prostate cancer Stomach-ache
*Rhus natalensis*	Busanguli	Desert date	Anacardiaceae	Indigenous	Shrub	Worm infections in humans and livestock
*Sapium ellypticum*	Musasa	Jumping seed tree	Euphorbiaceae	Indigenous	Tree	Eye problems in livestock such as by injury or infection
*Senecio moorei*	Not established	Not established	Asteraceae	Indigenous	Shrub	Cough
*Solanum incanum*	Indalandalwa	Sodom Apple	Solanaceae	Indigenous	Shrub	Stomach-ache
*Thunbergia alata*	Indereresia	Black-eyed Susan vine	Acanthaceae	Indigenous	Shrub	Joint dislocation in both humans and livestock
*Toddalia asiatica*	Not established	Orange climber	Rutaceae	Indigenous	Shrub	Worms in cattle
*Trichilia emetica*	Munyama	Banket mahogany	Meliaceae	Indigenous	Tree	Fever Stomach-ache Sexually transmitted infections Malaria
*Zanthoxyllum gilleti*	Shikhoma	Not established	Rutaceae	Indigenous	Tree	Cough and chest complications associated with bacterial infection

## Results and discussion

A total of 40 species of medicinal plants used by the people around the Kakamega forest were identified and recorded ( Table 1 and data set). The species fall into 25 families ( Table 2) and the list represents 11% of all plant species recorded in Kakamega forest ^21^. It certainly not presumed here that the list of species from this study is a complete one for the Kakamega forest as, due to the constraints of time and resources, the study did not cover every part the forest. The most dominant families were Asteraceae, Fabaceae and Lamiaceae, each representing 10.3% of all species collected.

**Table 2.** Families and corresponding number of species of medicinal plants identified.

Family	No of species	% proportion (N = 40)
Acanthaceae	2	5
Anacardiaceae	1	2.5
Apocynaceae	1	2.5
Asteraceae	4	10.3
Bignoniaceae	1	2.5
Ebenaceae	1	2.5
Euphorbiaceae	2	5
Fabaceae	4	10.3
Flacourtiaceae	1	2.5
Lamiaceae	4	10.3
Leguminoceae	1	2.5
Malvaceae	1	2.5
Melastomataceae	1	2.5
Meliaceae	2	5
Oleaceae	1	2.5
Paulowniaceae	1	2.5
Piperaceae	2	5
Ranunculaceae	1	2.5
Rosaceae	1	2.5
Rubiaceae	1	2.5
Rutaceae	2	5
Sapindaceae	1	2.5
Sapotaceae	1	2.5
Solanaceae	1	2.5
Verbenaceae	2	5

Medicinal plant species identified in and around Kakamega forest Profiles of 40 putative medicinal plant species identified in and around Kakamega forest

Of the 40 species, 22 were shrubs, 13 trees, 3 lower plants such as herbs or forbs, and 2 were climbers. This dominance of the shrubs also supports the prominence of the three families of Asteraceae, Fabaceae and Lamiaceae ( Table 2). Twenty-six of the medicinal species occurred inside the forest itself and 14 occurred outside. One of the species *(Prunus africana)* is also listed in the IUCN Red List as vulnerable to extinction ^22^. This species was encountered inside the forest while no other such threatened species was encountered outside the forest and this might underscore the forest reserve’s role in aiding the conservation of medicinal species.

The majority of the species identified (95%) were indigenous and only 5% were exotic ( Table 1) a fact that also reflects the localized nature of the indigenous knowledge about these medicinal plant species. For instances, despite the presence of *Eucalyptus sp* (family Myrtaceae) and *Grevillea sp* (family Proteaceae) in and around certain parts of the forest such as the Isecheno and Buyangu blocks, no informant mentioned any medicinal uses associated with them. Some *Eucalyptus* species are known to be used in treatment of certain bacterial or fungal infections in humans ^23^ while *Greville sp* is used in treatment of skin sores and as an antiseptic ^24^.

The total number of species recorded in this study compares closely to that recorded by Jeruto ^9^ in a study of medicinal plants used around the Nandi forest but is much smaller than the 107 species recorded in a study by Okello *et al.* for medicinal plants used by the Sabaot people around Mt. Elgon ^10^ and the 119 species recorded by Ndegwa of medicinal plants used by the Ogiek people in the East Mau forest ^11^.

**Figure 2.**
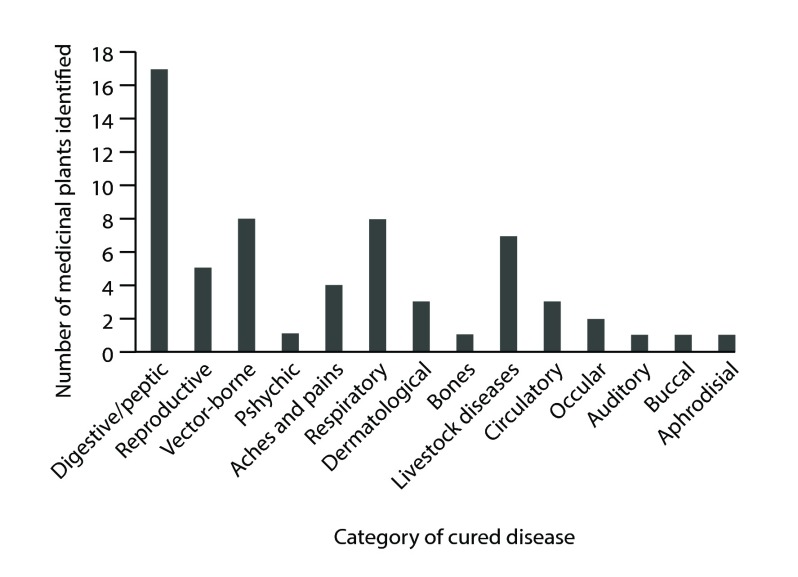
Overall distribution of categories of disease treated using medicinal plants recorded.

The diseases reported to be treated using the plant species varied widely but were grouped into 14 categories including use in the treatment of a number of livestock diseases ( Figure 2). Ninety percent of the diseases treated are those that affect humans and about ten percent for livestock diseases. Most of the human diseases treated using these species, fell into the categories of digestive or peptic; respiratory, vector-borne; and reproductive ailments ( Figure 2). Furthermore, these treatments are applicable for both genders and almost all age groups except in 17% of the cases where the treatments are applicable to adults only and 7% of the cases where treatments were applicable for old people only. 37% of the species are used by the local people to treat more than one condition. One particular species *Azadirachta indica* ( Table 1) is used by the local people to treat up to 6 different conditions, using all of its parts. This makes it the most valuable medicinal species even though it is of exotic origin ^7^. In 17% of the species, more than one plant part is used in the treatment of various conditons, not necessarily in combination.

**Figure 3.**
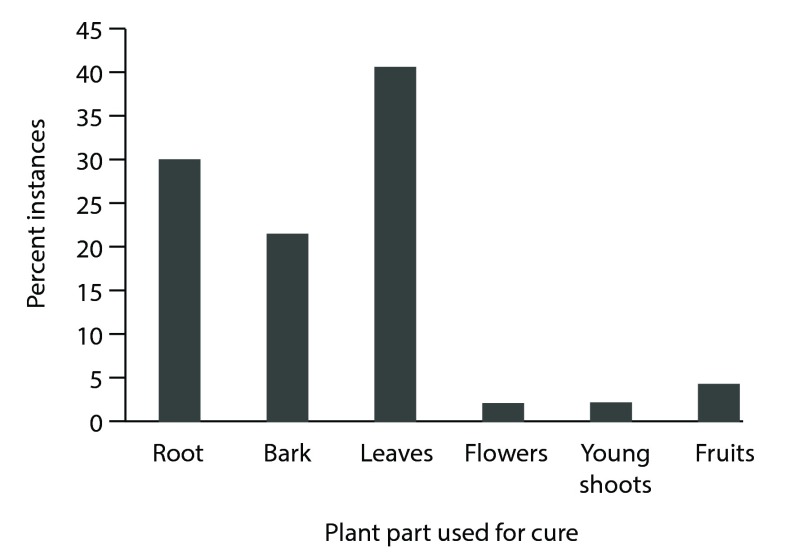
Percentage of use of the various parts of the medicinal plant species identified.

In preparing the treatments from the plants, the local people mainly use leaves, roots and barks, but in a few species, the treatment is derived from flowers, fruits and young shoots ( Figure 3). Additionally, since many of the species are used in treating digestive or peptic, respiratory or vector-borne ailments, the majority of them are administered orally as an infusion, concoction, decoction or a lick of its powdered form ^10^. The rest are applied either on the surface of the affected part of the body, through steam treatment, as fluid drops or through inhalation of either its fresh form or powder prepared from its crushed form.

A number of diseases are treated by many medicinal species, reflecting the prevalence of those specific conditions in the community. These included diseases related to stomach upsets (12 species), boils (2 species), fevers and aches (5 species), diarrhea (3 species), colds and flu (2 species), worm infestation (3 species) and malaria (4 species).

The results of this study demonstrate that apart from the Kakamega forest’s reputation as a significant Kenyan rainforest in terms of its rich biodiversity, eco-system service provision and as a remarkable tourist site ^7, 12– 14^, it is also important to the local community as a repository for ethno-pharmacological resources that play a crucial role in supplementing the government’s effort in human and veterinary healthcare at the grass-root level, like the neighboring Nandi forest ^9^. Much of the indigenous knowledge about these plant-based remedies is still held mainly by elderly members of the community. Furthermore, most knowledge holders tend to descend from families with long histories of the practice of traditional herbal healing.

In-depth discussions with the informants and a cross-section of some respondents among the local residents further revealed that even when the healers prescribe treatment to their patients, only the ready-made preparations are provided by the traditional healers meaning the patients would not be informed of the plant species from which the treatment is derived nor the method of preparation of the treatment. Nevertheless, this system is slowly changing and in recent years, some flexibility appears to be emerging, with the traditional healers, including the ones interviewed in this study, quite willing to provide information about the traditional treatments in exchange for financial inducement or compensation. For example, it is not uncommon to see young people hawking such easily used medicinal plants as *Mondia whytei* (see Table 1) along the streets of local urban areas. Such financial inducement was reported by the informants as a motivation for a growing crop of up-coming but semi-skilled traditional healers in the community.

Although this study was concerned with the wide variety of diseases treated using the medicinal plant species found in the Kakamega forest, the percentage proportions of medicinal plant types (shrubs, trees, herbs, climbers and lianas) is similar to that found by Jeruto *et al.* ^25^ who carried out a similar but narrower study in the Nandi forest for species used in treatment of malaria only. This latter study identified 40 medicinal plant species just like in our study, perhaps because of the larger spatial coverage of their study area.

In terms of plant parts used in treatment, leaves were predominantly used ( Figure 3). This concurs with findings of a study in south-western Ethiopia ^26^ and in Morogoro, Tanzania ^27^, although these comparative studies were not carried out in forest habitats. However, it differs from findings of a similar survey conducted in the Mau forest, Kenya ^11^ and in Mt. Elgon ^10^, in both cases the use of roots was found to be predominant. One presumption for prominent use of leaves for treatments in Kakamega is that the destructive methods associated with root or bark harvesting, is restricted or not permissible or compatible with the conservation policies for the Kakamega ^17^ forest where most of the species are derived. Thus, extracting leaves provides a more sustainable use strategy through rapid replacement by re-growth and is a practice acquired down the generations ^28^. Leaves are also easier to harvest and prepare into various concoctions, decoctions or infusions such as an express juice for administration in treatment, than roots and bark. In addition, the preparation of various extracts from leaves ensures better preservation of the active ingredients of the medication, that in the case of other parts of the plant ^10^.

According to the respondents, most of the treatments are administered orally either as infusions, decoctions or concoctions. Similar results were obtained in another earlier study by Jeruto *et al.* in South Nandi forest specifically ^9^. This appears to be consistent with the fact that most of them are used to treat diseases related to the digestive, oral tract or respiratory system ( Figure 2). The high prevalence of digestive and respiratory-related diseases, compared to other afflictions, also appears to reflect relatively poor sanitation due to the high density of human population in the district (433 persons per kilometre ^17^), an area that relies on only one main public healthcare facility, the Kakamega Municipal Hospital ^29^. The mean distance is 10 km from patient-to-hospital and the doctor to patient ratio is 1:14,200. This is compounded by a poverty level of 52% and increasing levels of school drop-outs ^29^, implying correspondingly diminished knowledge about basic health and sanitation which are essential in managing such communicable digestive or respiratory diseases.

## Conclusion

In conclusion, there is sufficient indigenous knowledge among the community around the Kakamega forest about medicinal plant species, to contribute not only to a sustainable provision of grass-root health care but also a potential to share this knowledge beyond western Kenya. Much of this knowledge is still held mainly by a few elderly people though financial inducements are said to be motivating a growing interest in the acquisition of knowledge among the wider community about these medicinal plants. This is encouraging because as the cost of conventional modern healthcare continues to increase, pushing such services out of reach to most rural dwellers in developing countries ^30^, there is a corresponding increased need to identify more affordable alternatives for the treatment of many ailments that affect rural populations. Unlocking such knowledge from the monopoly of a few to the wider population through an “accelerated” social construction ^31^ process such as through sustained public awareness campaigns, story telling or role plays, should thus be encouraged because such indigenous knowledge also has a potential for boosting economic empowerment of the local people through the sale of intellectual property rights or social capital. This may be leveraged further to boost conservation of such habitats from which medicinal plants are sourced, such as forests.

## Recommendations

- More extensive excursions into the Kakamega forest and its immediate surroundings to reveal more medicinal plant species, particularly through the involvement of a larger number of key informants. Low numbers of informants were used because our study was constrained by time and logistical issues, thus not allowing us to cover the whole forest. As evident, the total number of medicinal plants identified is unexpectedly small in comparison, for instance, to similar areas such as the South Nandi forest ^26^. Also it would be interesting to see if an equal number of male and female informants in the study might yield different knowledge perspectives such as the dominance of diseases of the alimentary canal and use of leaves over other medicinal plant parts, in the treatment of various diseases.
- A deliberate effort to make accessible results of earlier studies on medicinal plants of the Kakamega forest, anecdotal and otherwise, would make such knowledge more widely accessible to the wider public for use in the treatment of diseases. This could be through publishing, with technical review and support involving local and scientific stakeholders.
- Promotion of the use of natural remedies derived from various locally based resources such as medicinal plant species, should form an important priority of the Kenyan governments’ strategies to make healthcare accessible to rural populations in a more affordable way.
