# Supplementary material for: Local indigenous knowledge about some medicinal plants in and around Kakamega forest in western Kenya
Source: F1000Res. 2012 Dec 13;1:40. Originally published 2012 Oct 31. [Version 2] doi: 10.12688/f1000research.1-40.v2 (PMC3954169; doi:10.12688/f1000research.1-40.v2)
Supplement: Questionnaire provided to local informants to identify local medicinal plants — The questionnaire provided to local informants to identify local medicinal plants [file f1000research-1-603-s0001.tgz › Questionnaire.pdf]

Local indigenous knowledge about medicinal plants in and around Kakamega forest in western Kenya (Nickson Otieno et al.)

Structured Questionnaire

Preliminary questions

- Respondent name:………………………………………………………….
- Respondent age:……………………….
- Respondent total duration or residence around study area:………years

Plant Attributes

- Local name: ……………………………….

- Plant form (tick as appropriate. Filled in by interviewer from observation):
  Tree………Shrub…………Herb………Forbe…………Sedge………Grass……C
  limber……………..Other(specify)……………………

- Plant origin (tick as appropriate):
  Indigenous…………..Exotic)………………………..

- Common (English) name: to be filled in by interviewer
  ……………………………………………………………

- Family: (to be filled in by interviewer):……………………………..

- Scientific name (to be filled in by
  interviewer):…………………………………………………………………

Collection site

- In relation to forest (Tick as appropriate)
  Inside………….Outside):…………………….

- Forest block name: ………………………………………

Symptoms or condition cured (List)

1. …………………………………………………………………………
2. …………………………………………………………………………
3. …………………………………………………………………………
4. …………………………………………………………………………
5. …………………………………………………………………………

Collection site description
……………………………………………………………………………………………………
……………………………………………………………………………………………………

Part(s) used or from which medicine is extracted (tick as appropriate)
Leaves ……………………………………….
Bark ……………………………………….
Roots ……………………………………….
Fruits ……………………………………….
Flowers ……………………………………….
Other (specify)
……………………………………………………………………………………………………
……………………………………………………………………………………………………

General preparation method
……………………………………………………………………………………………………
……………………………………………………………………………………………………
……………………………………………………………………………………………………

Method of administering medication
……………………………………………………………………………………………………
……………………………………………………………………………………………………
……………………………………………………………………………………………………
……………………………………………………………………………………………………
……………………………………………………………………………………………………

Patient age group (tick as appropriate)
Elderly ……………………………………….
Adults……………………………………….
Youth……………………………………….
Children……………………………………….
Infants……………………………………….
All age groups…………………………….

Patient gender (tick as appropriate)
Male ……………………………………….
Female……………………………………….
Both genders …………………………….

Post-field open-ended focused discussion

- Who possesses the general knowledge about these medicinal plants?
- Are there other beneficiaries of stakeholders to this indigenous knowledge other than the people around Kakamega forest?
- Any other information you may want to add?
