# Supplementary material for: Local indigenous knowledge about some medicinal plants in and around Kakamega forest in western Kenya
Source: F1000Res. 2012 Dec 13;1:40. Originally published 2012 Oct 31. [Version 2] doi: 10.12688/f1000research.1-40.v2 (PMC3954169; doi:10.12688/f1000research.1-40.v2)
Supplement: Medicinal plant species identified in and around Kakamega forest — Profiles of 40 putative medicinal plant species identified in and around Kakamega forest [file f1000research-1-603-s0000.tgz › Albizia_grandi_bracteata.pdf]

Albizia grandi bracteata

Attributes

- Local name: Mukhunzuli
- Common name: Large-leaved Albizia
- Family: Fabaceae
- Plant origin: Indigenous
- Plant form: Tree

Collection site

- In relation to forest: Outside forest
- Forest block: Buyangu
- Specific site name: Kisere

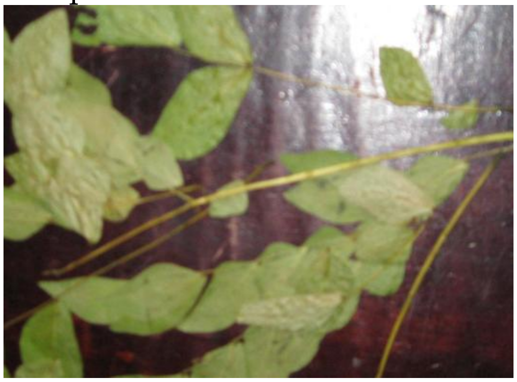

Symptoms or condition cured
Gonorrhea

Collection site description
Natural (undisturbed) area at forest edge

Part used/from which medicine is extracted
Bark

General preparation method
Bark boiled in water till it turns brown

Method of administering medication
The extract is taken cold in the mornings and evenings for seven days consecutively

Patient age group
Useful for all age groups

Patient gender: Both genders
