# Supplementary material for: Local indigenous knowledge about some medicinal plants in and around Kakamega forest in western Kenya
Source: F1000Res. 2012 Dec 13;1:40. Originally published 2012 Oct 31. [Version 2] doi: 10.12688/f1000research.1-40.v2 (PMC3954169; doi:10.12688/f1000research.1-40.v2)
Supplement: Medicinal plant species identified in and around Kakamega forest — Profiles of 40 putative medicinal plant species identified in and around Kakamega forest [file f1000research-1-603-s0000.tgz › Albizia_gummifera.pdf]

Albizia gummifera

Attributes

- Local name: Musenzeli
- Common name: Peacock flower
- Family: Fabaceae
- Plant origin: Indigenous
- Plant form: Tree

Collection site

- In relation to forest: Inside
- Forest block: Isecheno
- Specific site name: Muleche

Collection site description

Farmland

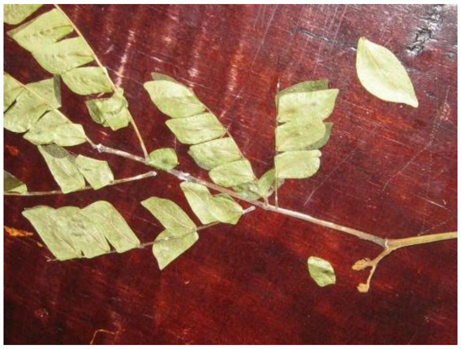

Symptoms or condition cured

- Sexually transmitted infections especially gonorrhea;
- Stomach-ache

Part used/from which medicine is extracted

Roots and bark

General preparation method

Roots and bark crashed and boiled

Method of administering medication

The concoction taken orally when cool, daily till healing is achieved

Patient age group

Useful for adults

Patient gender: Both genders
