# Supplementary material for: Local indigenous knowledge about some medicinal plants in and around Kakamega forest in western Kenya
Source: F1000Res. 2012 Dec 13;1:40. Originally published 2012 Oct 31. [Version 2] doi: 10.12688/f1000research.1-40.v2 (PMC3954169; doi:10.12688/f1000research.1-40.v2)
Supplement: Medicinal plant species identified in and around Kakamega forest — Profiles of 40 putative medicinal plant species identified in and around Kakamega forest [file f1000research-1-603-s0000.tgz › Aspilia_pluriseta.pdf]

Aspilia pluriseta
Attributes
- Local name: Shralambila
- Common name: Dwarf aspilia
- Family: Asteraceae
- Plant origin: Indigenous
- Plant form: Herb/forb

Collection site
- In relation to forest: Outside
- Forest block: Ikuywa
- Specific site name: Ikuywa

Collection site description
Human area
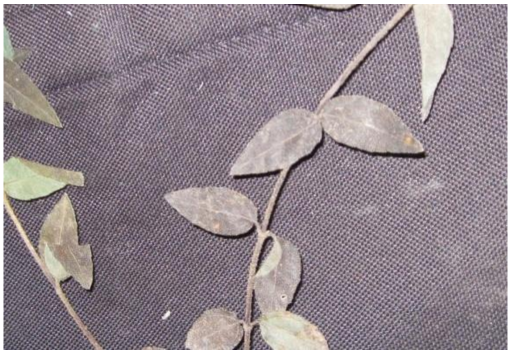
Symptoms or condition cured
- Stopping bleeding in wounds;
- Drippy nose in poultry

Part used/from which medicine is extracted
Leaves for both

General preparation method
- For stopping bleeding, leaves are crushed firmly between hands
- For poultry nose cure, leaves are crushed and in mixture with water
-

Method of administering medication
- To stop bleeding, the crushed leaves are applied by pressing firmly onto wound, holding on till bleeding stops
- For affected poultry, the extract is administered twice daily till symptoms disappear

Patient age group
All age groups
Patient gender: Both genders
