# Supplementary material for: Local indigenous knowledge about some medicinal plants in and around Kakamega forest in western Kenya
Source: F1000Res. 2012 Dec 13;1:40. Originally published 2012 Oct 31. [Version 2] doi: 10.12688/f1000research.1-40.v2 (PMC3954169; doi:10.12688/f1000research.1-40.v2)
Supplement: Medicinal plant species identified in and around Kakamega forest — Profiles of 40 putative medicinal plant species identified in and around Kakamega forest [file f1000research-1-603-s0000.tgz › Azadirachta_indica.pdf]

Azadirachta indica

Attributes

Local Name: Muarubaini (meaning a cure for 40 illnesses)

- Common Name: Neem tree
- Family: Meliaceae
- Plant origin: Exotic
- Plant form: Tree

Collection site
- In relation to forest: Outside
- Forest block: Kaimosi
- Specific site name: Kaptik

Collection site description
On farmland

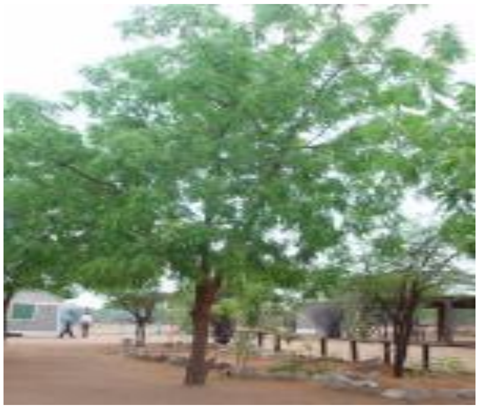

Symptoms or condition cured
Fever, aches, pains, malaria attack, insect bites, pest control, skin infections

Part used/from which medicine is extracted
Leaves, roots or bark

General preparation method
- For fever, malaria, aches and pains, the leaves boiled in water and the resulting dark extract filleted out
- For insect bites and skin infections, the fruits are pounded and the oil extracted
- For pest control, the leaves, roots or barks are dried and ground into powder

Method of administering medication
- For aches and pains, malaria and fever, the extracted filtrate is drunk while hot t least twice each day till symptoms disappear, or the patient is covered in a blanket over the steaming liquid for about 6 minutes twice each day

- For insect bites, skin infections and painful joints, the extracted oily substance from the seeds is applied by rubbing firmly and directly onto the wound or painful joints
- For pest control, the powder from the dried leaves or bark if spread over the infested area, or as in the case of domestic insects, the fresh leaves are smoke-burned in the infested room with most openings closed for at least 10 minutes

Patient age group
Any age group

Patient gender: Both genders
