# Supplementary material for: Local indigenous knowledge about some medicinal plants in and around Kakamega forest in western Kenya
Source: F1000Res. 2012 Dec 13;1:40. Originally published 2012 Oct 31. [Version 2] doi: 10.12688/f1000research.1-40.v2 (PMC3954169; doi:10.12688/f1000research.1-40.v2)
Supplement: Medicinal plant species identified in and around Kakamega forest — Profiles of 40 putative medicinal plant species identified in and around Kakamega forest [file f1000research-1-603-s0000.tgz › Bequartiodendron_oblanceolata.pdf]

Bequartiodendron oblanceolata

Attributes
- Local name: Musamia
- Common name: Not ascertained
- Family: Not clearly ascertained
- Plant origin: Indigenous
- Plant form: Tree

Collection site
- In relation to forest: Inside
- Forest block: Kaimosi
- Specific site name: Shinu

Collection site description
Natural (minimum-disturbance) area
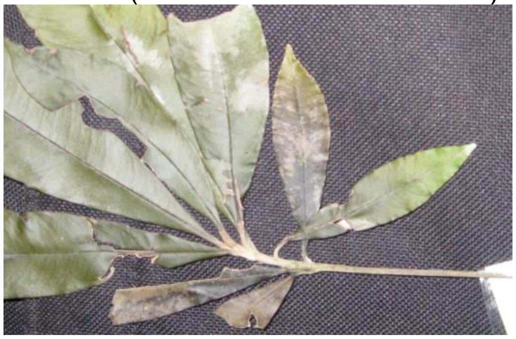
Symptoms or condition cured
Ulcers in digestive track
Boils especially around the belly

Part used/from which medicine is extracted
- Bark for ulcers;
- Leaves for boils

General preparation method
- For ulcers, bark boiled in water
- For boils, leaves are crushed in mixture with water

Method of administering medication
- For ulcers, the concoction taken by patient when it is cold
- For boils, the concoction is drunk daily till symptoms disappear

Patient age group
All age-groups older than 2 years

Patient gender: Both genders
