# Supplementary material for: Local indigenous knowledge about some medicinal plants in and around Kakamega forest in western Kenya
Source: F1000Res. 2012 Dec 13;1:40. Originally published 2012 Oct 31. [Version 2] doi: 10.12688/f1000research.1-40.v2 (PMC3954169; doi:10.12688/f1000research.1-40.v2)
Supplement: Medicinal plant species identified in and around Kakamega forest — Profiles of 40 putative medicinal plant species identified in and around Kakamega forest [file f1000research-1-603-s0000.tgz › Chrysocephalum_sp.pdf]

Chrysocephalum sp
Attributes
- Local name: Mwikalo
- Common name: Yellow Buttons
- Family: Asteraceae
- Plant origin: Indigenous
- Plant form: Shrub

Collection site
- In relation to forest: Inside
- Forest block: Ikuywa
- Specific site name: Chepsonoi

Collection site description
Natural (minimum-impact) area

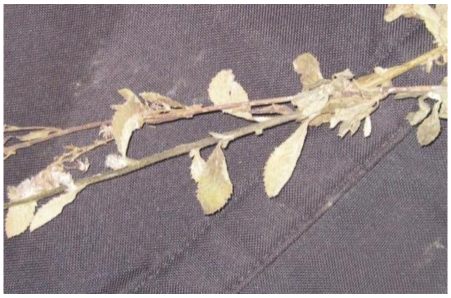

Symptoms or condition cured
Stomach problems especially abdominal pains and related sexually transmitted infections

Part used/from which medicine is extracted
Roots

General preparation method
Roots are chopped and boiled in water till dark brownish in colour

Method of administering medication
One glass of the concoction is taken orally twice each day (morning and evening)

Patient age group
Adults

Patient gender: Both genders
