# Supplementary material for: Local indigenous knowledge about some medicinal plants in and around Kakamega forest in western Kenya
Source: F1000Res. 2012 Dec 13;1:40. Originally published 2012 Oct 31. [Version 2] doi: 10.12688/f1000research.1-40.v2 (PMC3954169; doi:10.12688/f1000research.1-40.v2)
Supplement: Medicinal plant species identified in and around Kakamega forest — Profiles of 40 putative medicinal plant species identified in and around Kakamega forest [file f1000research-1-603-s0000.tgz › Clematopsis_scabiosifolia.pdf]

Clematopsis scabiosifolia

Attributes

- Local name: Lunyili
- Common name: Not ascertained
- Family: Ranunculaceae
- Plant origin: Indigenous
- Plant form: Climber

Collection site

- In relation to forest: Outside
- Forest block: Ikuywa
- Specific site name: Mukhumu

Collection site description: Farmland area 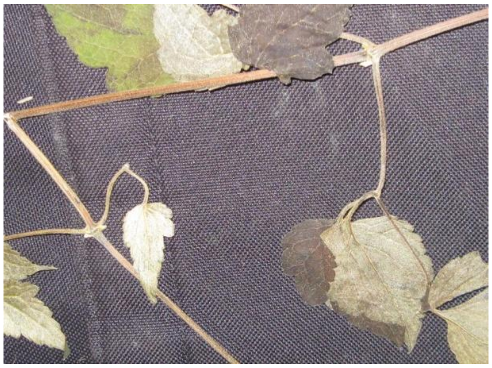

Symptoms or condition cured
Stuffy nose and associated respiratory problems

Part used/from which medicine is extracted
Flower

General preparation method
Young leaves are crushed between the hands

Method of administering medication
The crushed mass is inhaled at frequent regular intervals

Patient age group
All age groups above 3 years

Patient gender: Both genders
