# Supplementary material for: Local indigenous knowledge about some medicinal plants in and around Kakamega forest in western Kenya
Source: F1000Res. 2012 Dec 13;1:40. Originally published 2012 Oct 31. [Version 2] doi: 10.12688/f1000research.1-40.v2 (PMC3954169; doi:10.12688/f1000research.1-40.v2)
Supplement: Medicinal plant species identified in and around Kakamega forest — Profiles of 40 putative medicinal plant species identified in and around Kakamega forest [file f1000research-1-603-s0000.tgz › Clerodendron_pygmaeum.pdf]

Clerodendron pygmaeum

Attributes

- Local name: Luseshe
- Common name: Cashmere Bouquet/Strong-scented glorybower

- Family: Verbenaceae
- Plant origin: Indigenous
- Plant form: Shrub
- **Collection site**
- In relation to forest: Inside
- Forest block: Kaimosi
- Specific site name: Musasa

Collection site description: Natural (minimum-disturbance) area in a forest glade

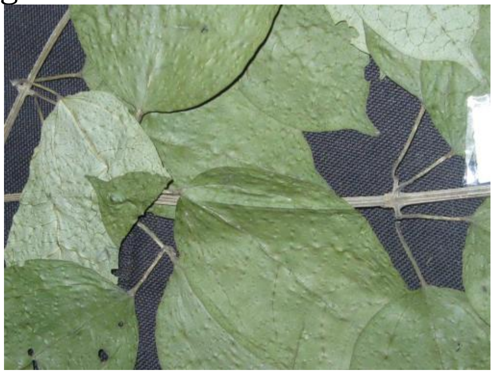

Symptoms or condition cured
Common flu and associated symptoms

Part used/from which medicine is extracted
Root

General preparation method
The roots are crushed

Method of administering medication
Crushed roots mix with water and taken frequently

Patient age group
All age groups

Patient gender: Both genders
