# Supplementary material for: Local indigenous knowledge about some medicinal plants in and around Kakamega forest in western Kenya
Source: F1000Res. 2012 Dec 13;1:40. Originally published 2012 Oct 31. [Version 2] doi: 10.12688/f1000research.1-40.v2 (PMC3954169; doi:10.12688/f1000research.1-40.v2)
Supplement: Medicinal plant species identified in and around Kakamega forest — Profiles of 40 putative medicinal plant species identified in and around Kakamega forest [file f1000research-1-603-s0000.tgz › Coffea_eugenioides.pdf]

Coffea eugenioides

Attributes

- Local name: Itikwa
- Common name: Mufindi coffee
- Family: Rubiaceae
- Plant origin: Indigenous
- Plant form: Shrub

Collection site

- In relation to forest: Inside
- Forest block: Kaimosi
- Specific site name: Musasa

Collection site description: Natural (minimum-disturbance) area

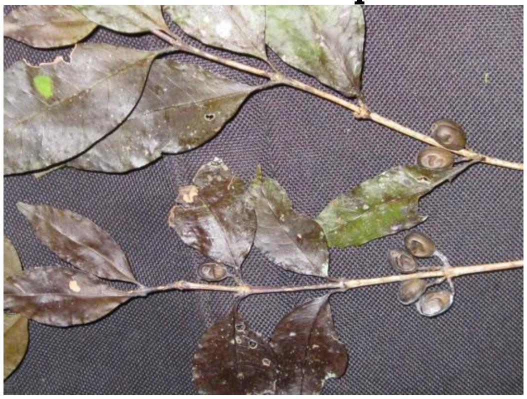

Symptoms or condition cured
Eye problems in livestock

Part used/from which medicine is extracted
Leaves
General preparation method
Leaves are crushed

Method of administering medication
Crushed leaves mixed with water and exudate sqeeze-dropped into affected eye(s) frequently

Patient age group
N/A

Patient gender: N/A
