# Supplementary material for: Local indigenous knowledge about some medicinal plants in and around Kakamega forest in western Kenya
Source: F1000Res. 2012 Dec 13;1:40. Originally published 2012 Oct 31. [Version 2] doi: 10.12688/f1000research.1-40.v2 (PMC3954169; doi:10.12688/f1000research.1-40.v2)
Supplement: Medicinal plant species identified in and around Kakamega forest — Profiles of 40 putative medicinal plant species identified in and around Kakamega forest [file f1000research-1-603-s0000.tgz › Conyza_floribunda.pdf]

Conyza floribunda

Collection site
In relation to forest: Outside
Forest block: Ikuywa
Specific site name: Mukhumu

Collection site description
Area with some human settlement

Attributes
- Local name: Liposhe
- Common name: Asthma weed
- Family: Asteraceae
- Plant origin: Indigenous
- Plant form: Shrub

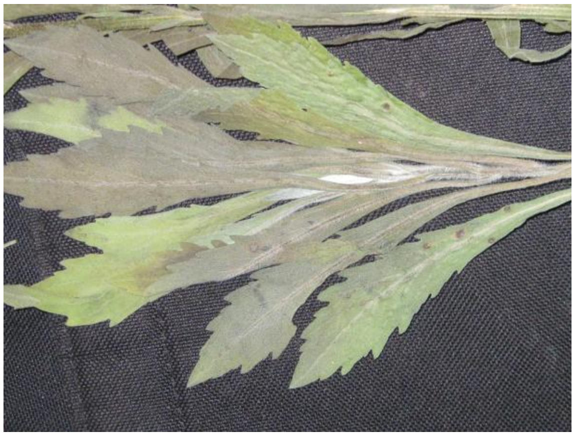

Symptoms or condition cured
Tooth-ache

Part used/from which medicine is extracted
Leaves

General preparation method
Leaves are harvested and cleaned with water

Method of administering medication
The patient chews the leaves especially involving the affected tooth-teeth

Patient age group
All age groups above 2 years

Patient gender: Both genders
