# Supplementary material for: Local indigenous knowledge about some medicinal plants in and around Kakamega forest in western Kenya
Source: F1000Res. 2012 Dec 13;1:40. Originally published 2012 Oct 31. [Version 2] doi: 10.12688/f1000research.1-40.v2 (PMC3954169; doi:10.12688/f1000research.1-40.v2)
Supplement: Medicinal plant species identified in and around Kakamega forest — Profiles of 40 putative medicinal plant species identified in and around Kakamega forest [file f1000research-1-603-s0000.tgz › Desmodium_adscendens.pdf]

Desmodium adscendens

Attributes

- Local name: Matite
- Common name: Not ascertained
- Family: Fabaceae
- Plant origin: Indigenous
- Plant form: Herb/forb

Collection site

- In relation to forest: Inside forest
- Forest block: Buyangu
- Specific site name: Kisere

Collection site description
Natural (undisturbed) area

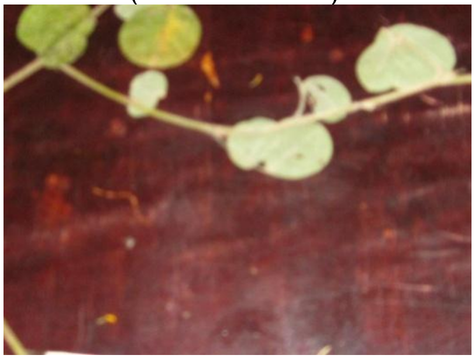

Symptoms or condition cured
Stomach-ache

Part used/from which medicine is extracted
Leaves

General preparation method
Crush leaves and mix with water

Method of administering medication
The mixture is taken cold particularly after a meal

Patient age group
Useful for all age groups

Patient gender: Both genders
