# Supplementary material for: Local indigenous knowledge about some medicinal plants in and around Kakamega forest in western Kenya
Source: F1000Res. 2012 Dec 13;1:40. Originally published 2012 Oct 31. [Version 2] doi: 10.12688/f1000research.1-40.v2 (PMC3954169; doi:10.12688/f1000research.1-40.v2)
Supplement: Medicinal plant species identified in and around Kakamega forest — Profiles of 40 putative medicinal plant species identified in and around Kakamega forest [file f1000research-1-603-s0000.tgz › Desmodium_repandum.pdf]

Desmodium repandum
Attributes
- Local name: Not ascertained
- Common name: Not ascertained
- Family: Fabaceae
- Plant origin: Indigenous
- Plant form: Shrub

Collection site
- In relation to forest: Inside forest
- Forest block: Buyangu
- Specific site name: Shanderema

Collection site description
Natural (undisturbed) area

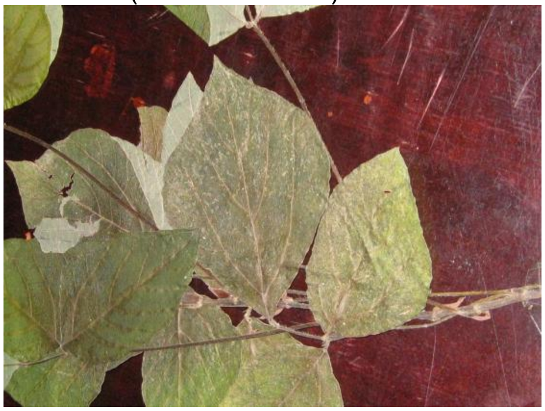

Symptoms or condition cured
Stomach upset

Part used/from which medicine is extracted
Leaves

General preparation method
Crush leaves in mixture with water

Method of administering medication
The mixture is taken orally

Patient age group
Useful for all age groups

Patient gender: Both genders
