# Supplementary material for: Local indigenous knowledge about some medicinal plants in and around Kakamega forest in western Kenya
Source: F1000Res. 2012 Dec 13;1:40. Originally published 2012 Oct 31. [Version 2] doi: 10.12688/f1000research.1-40.v2 (PMC3954169; doi:10.12688/f1000research.1-40.v2)
Supplement: Medicinal plant species identified in and around Kakamega forest — Profiles of 40 putative medicinal plant species identified in and around Kakamega forest [file f1000research-1-603-s0000.tgz › Diospyros_abyssinica.pdf]

Diospyros abyssinica
Attributes
- Local name: Lusui
- Common name: Giant Ebony
- Family: Ebenaceae
- Plant origin: Indigenous
- Plant form: Tree

Collection site
- In relation to forest: Inside
- Forest block: Isecheno
- Specific site name: Virembe

Collection site description
Natural (undisturbed) area

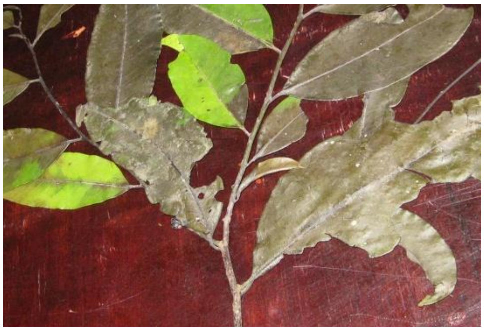

Symptoms or condition cured
- Recurrent nightmares
- Sores

Part used/from which medicine is extracted
- For nightmares, the bark;
- For sores, leaves

General preparation method
- For nightmares, bark used in bedroom
- For sores, fresh leaves are crushed

Method of administering medication
- For nightmares, bark placed under pillow or close to head as victim goes to sleep
- For sores, the crushed leaves are squeezed into the wound daily

Patient age group
Useful mainly for adults
Patient gender: Both genders
