# Supplementary material for: Local indigenous knowledge about some medicinal plants in and around Kakamega forest in western Kenya
Source: F1000Res. 2012 Dec 13;1:40. Originally published 2012 Oct 31. [Version 2] doi: 10.12688/f1000research.1-40.v2 (PMC3954169; doi:10.12688/f1000research.1-40.v2)
Supplement: Medicinal plant species identified in and around Kakamega forest — Profiles of 40 putative medicinal plant species identified in and around Kakamega forest [file f1000research-1-603-s0000.tgz › Dissotis_speciosa.pdf]

Dissotis speciosa

Attributes
- Local name: Lunyili
- Common name: Not ascertained
- Plant origin: Indigenous
- Plant form: Shrub

Collection site
- In relation to forest: Outside
- Forest block: Ikuywa
- Specific site name: Mukhumu

Collection site description
Farmland area

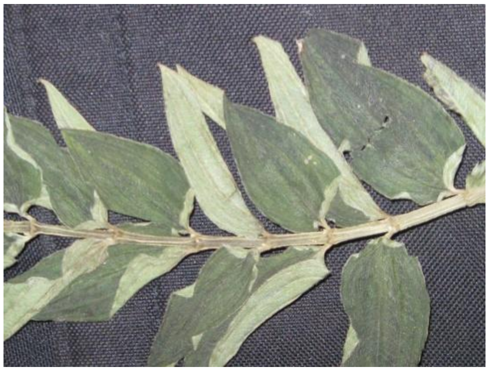

Symptoms or condition cured
Diarrhea

Part used/from which medicine is extracted
Leaves

General preparation method
Leaves are crushed in mixture with water

Method of administering medication
The patient takes the liquid extract orally

Patient age group
All age groups

Patient gender: Both genders
