# Supplementary material for: Local indigenous knowledge about some medicinal plants in and around Kakamega forest in western Kenya
Source: F1000Res. 2012 Dec 13;1:40. Originally published 2012 Oct 31. [Version 2] doi: 10.12688/f1000research.1-40.v2 (PMC3954169; doi:10.12688/f1000research.1-40.v2)
Supplement: Medicinal plant species identified in and around Kakamega forest — Profiles of 40 putative medicinal plant species identified in and around Kakamega forest [file f1000research-1-603-s0000.tgz › Dovyalis_macrocalyx.pdf]

Dovyalis macrocalyx

Attributes
- Local name: Shinavatevia
- Common name: Shaggy-fruited dovyalis
- Family: Flacourtiaceae
- Plant origin: Indigenous
- Plant form: Shrub

Collection site
- In relation to forest: inside
- Forest block: Buyangu
- Specific site name: Kisere

Collection site description: Natural (undisturbed) area

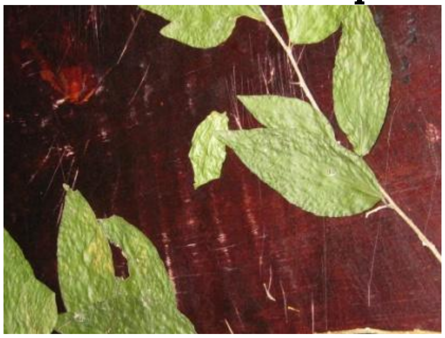

Symptoms or condition cured
- Constipation
- Peptic ulcers

Part used/from which medicine is extracted
- Roots for constipation
- Leaves for peptic ulcers

General preparation method
- For constipation, clean roots are smashed and stir-mixed with water
- For peptic ulcers, leaves are crushed in mixture with honey

Method of administering medication
- For constipation, extracted liquid taken orally by patient
- For peptic ulcers, the mixture is taken orally at least twice daily for a month or till symptoms disappear

Patient age group
Mostly elderly people

Patient gender: Both genders
