# Supplementary material for: Local indigenous knowledge about some medicinal plants in and around Kakamega forest in western Kenya
Source: F1000Res. 2012 Dec 13;1:40. Originally published 2012 Oct 31. [Version 2] doi: 10.12688/f1000research.1-40.v2 (PMC3954169; doi:10.12688/f1000research.1-40.v2)
Supplement: Medicinal plant species identified in and around Kakamega forest — Profiles of 40 putative medicinal plant species identified in and around Kakamega forest [file f1000research-1-603-s0000.tgz › Entada_abyssinica.pdf]

Entada abyssinica

Attributes
- Local name: Shivayamboga
- Common name: Abyssinia Entada
- Family: Leguminoceae
- Plant origin: Indigenous
- Plant form: Tree

Collection site
- In relation to forest: Outside
- Forest block: Ikuywa
- Specific site name: Chepsonoi

Collection site description
Farmland area

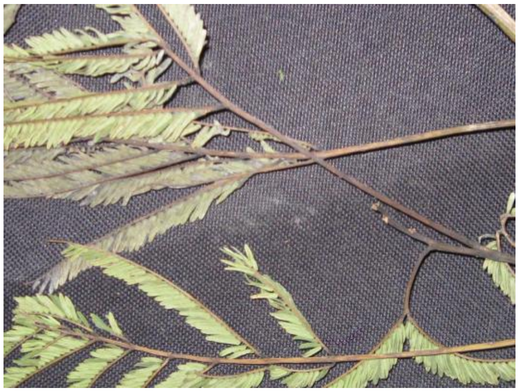

Symptoms or condition cured
Stomach-ache

Part used/from which medicine is extracted
Bark
General preparation method
Crush and boil the bark in water

Method of administering medication

The extract is orally taken

Patient age group
All age groups

Patient gender: Both genders
