# Supplementary material for: Local indigenous knowledge about some medicinal plants in and around Kakamega forest in western Kenya
Source: F1000Res. 2012 Dec 13;1:40. Originally published 2012 Oct 31. [Version 2] doi: 10.12688/f1000research.1-40.v2 (PMC3954169; doi:10.12688/f1000research.1-40.v2)
Supplement: Medicinal plant species identified in and around Kakamega forest — Profiles of 40 putative medicinal plant species identified in and around Kakamega forest [file f1000research-1-603-s0000.tgz › Erythrococca_atrovirens.pdf]

Erythrococca atrovirens

Attributes

- Local name: Shirietso
- Common name: Not ascertained
- Family: Euphorbiaceae
- Plant origin: Indigenous
- Plant form: Shrub

Collection site

- In relation to forest: inside
- Forest block: Buyangu
- Specific site name: Ivakale

Collection site description
Natural (undisturbed) area

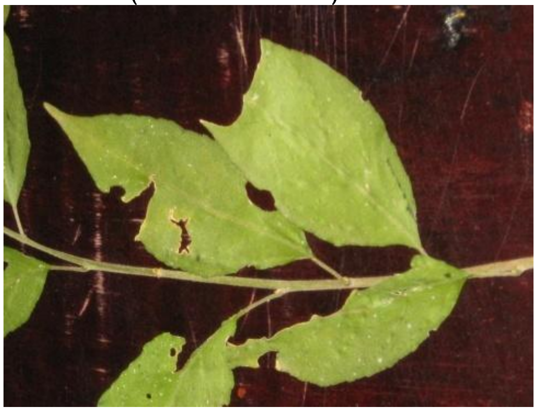

Symptoms or condition cured
Wounds, especially septic

Part used/from which medicine is extracted
Leaves

General preparation method
Leaves crushed firmly between hands

Method of administering medication
Extracted liquid from freshly crashed leaves is applied by directly pressing the crushed mass onto the wound

Patient age group
Mostly elderly people

Patient gender: Both genders
