# Supplementary material for: Local indigenous knowledge about some medicinal plants in and around Kakamega forest in western Kenya
Source: F1000Res. 2012 Dec 13;1:40. Originally published 2012 Oct 31. [Version 2] doi: 10.12688/f1000research.1-40.v2 (PMC3954169; doi:10.12688/f1000research.1-40.v2)
Supplement: Medicinal plant species identified in and around Kakamega forest — Profiles of 40 putative medicinal plant species identified in and around Kakamega forest [file f1000research-1-603-s0000.tgz › Hibiscus_sp.pdf]

Hibiscus sp

Attributes

- Local name: Lubulwa
- Common name: Not ascertained
- Family: Malvoideae
- Plant origin: Indigenous
- Plant form: Shrub

Collection site

- In relation to forest: Inside
- Forest block: Kaimosi
- Specific site name: Kaptik

Collection site description: Natural (minimum-disturbance) area in a glade

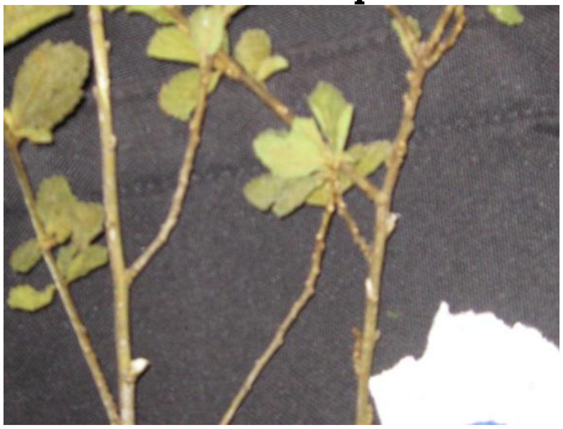

Symptoms or condition cured
Stomach-ache and general fever

Part used/from which medicine is extracted
Roots

General preparation method
Roots boiled in water

Method of administering medication
Extract taken either warm or cold. At least half a glass each time from first preparation then full glass each time for subsequent preparations of the same bark

Patient age group
All age groups except especially adults

Patient gender: Both genders
