# Supplementary material for: Local indigenous knowledge about some medicinal plants in and around Kakamega forest in western Kenya
Source: F1000Res. 2012 Dec 13;1:40. Originally published 2012 Oct 31. [Version 2] doi: 10.12688/f1000research.1-40.v2 (PMC3954169; doi:10.12688/f1000research.1-40.v2)
Supplement: Medicinal plant species identified in and around Kakamega forest — Profiles of 40 putative medicinal plant species identified in and around Kakamega forest [file f1000research-1-603-s0000.tgz › Justica_flava.pdf]

Justica flava

Attributes

- Local name: Lihululwa
- Common name: Yellow Justicea
- Family: Acanthaceae
- Plant origin: Indigenous
- Plant form: Herb/forb

Collection site

- In relation to forest: Inside
- Forest block: Ikuywa
- Specific site name: Ikuywa

Collection site description

Natural (minimum-impact) area

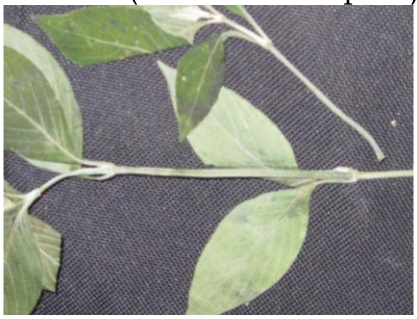

Symptoms or condition cured

Reducing post-natal pains

Part used/from which medicine is extracted

Roots

General preparation method

Roots are crushed in mixture with water

Method of administering medication

The mixture is taken orally by new mother immediately after delivery

Patient age group

Adults

Patient gender: Females
