# Supplementary material for: Local indigenous knowledge about some medicinal plants in and around Kakamega forest in western Kenya
Source: F1000Res. 2012 Dec 13;1:40. Originally published 2012 Oct 31. [Version 2] doi: 10.12688/f1000research.1-40.v2 (PMC3954169; doi:10.12688/f1000research.1-40.v2)
Supplement: Medicinal plant species identified in and around Kakamega forest — Profiles of 40 putative medicinal plant species identified in and around Kakamega forest [file f1000research-1-603-s0000.tgz › Lantana_trifolia.pdf]

Lantana trifolia

Attributes
- Local name: Imbulimutacha
- Common name: Three-leaf Shrub verbena
- Family: Verbenaceae
- Plant origin: Indigenous
- Plant form: Shrub

Collection site
- In relation to forest: Inside
- Forest block: Kaimosi
- Specific site name: Kaptik

Collection site description
Natural (minimum-disturbance) area in a glade

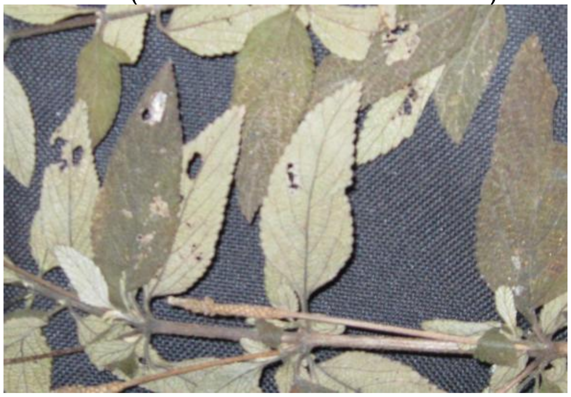

Symptoms or condition cured
Malaria and general fever (humans);
Diarrhea in livestock

Part used/from which medicine is extracted
Roots in both cases

General preparation method
In both cases, roots are crushed and mixed with water

Method of administering medication
- For malaria, the concoction taken orally when cold, twice daily
- For livestock, the concoction is administered each morning only, in cold form

Patient age group
For humans, all age groups except babies under 2 years

Patient gender: Both genders
