# Supplementary material for: Local indigenous knowledge about some medicinal plants in and around Kakamega forest in western Kenya
Source: F1000Res. 2012 Dec 13;1:40. Originally published 2012 Oct 31. [Version 2] doi: 10.12688/f1000research.1-40.v2 (PMC3954169; doi:10.12688/f1000research.1-40.v2)
Supplement: Medicinal plant species identified in and around Kakamega forest — Profiles of 40 putative medicinal plant species identified in and around Kakamega forest [file f1000research-1-603-s0000.tgz › Leucas_calostachys.pdf]

Leucas calostachys

Attributes
- Local name: Lumetsani
- Common name: Not ascertained
- Family: Lamiaceae
- Plant origin: Indigenous
- Plant form: Shrub

Collection site
- In relation to forest: Inside
- Forest block: Ikuywa
- Specific site name: Chepsonoi

Collection site description: Grassland (near-natural)

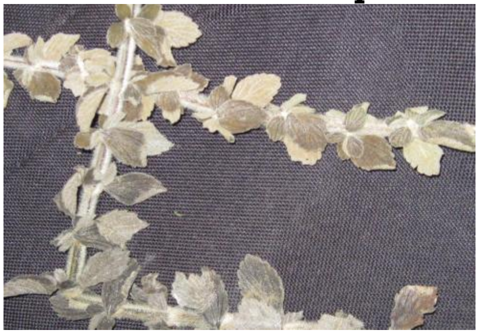

Symptoms or condition cured
Severe diarrhea especially accompanied with blood stains

Part used/from which medicine is extracted
Leaves

General preparation method
Leaves crushed and mixed with water

Method of administering medication
Extract taken orally by half a cup each morning and evening till diarrhea stops

Patient age group
All age groups above 2 years

Patient gender: Both genders
