# Supplementary material for: Local indigenous knowledge about some medicinal plants in and around Kakamega forest in western Kenya
Source: F1000Res. 2012 Dec 13;1:40. Originally published 2012 Oct 31. [Version 2] doi: 10.12688/f1000research.1-40.v2 (PMC3954169; doi:10.12688/f1000research.1-40.v2)
Supplement: Medicinal plant species identified in and around Kakamega forest — Profiles of 40 putative medicinal plant species identified in and around Kakamega forest [file f1000research-1-603-s0000.tgz › Markhamia_lutea.pdf]

Markhamia lutea
Attributes
- Local name: Lusiola
- Common name: Nile Tulip tree
- Family: Bignoniaceae
- Plant origin: Indigenous
- Plant form: Tree

Collection site
- In relation to forest: Inside
- Forest block: Kaimosi
- Specific site name: Shinu

Collection site description
Natural (minimum-disturbance) area near forest edge

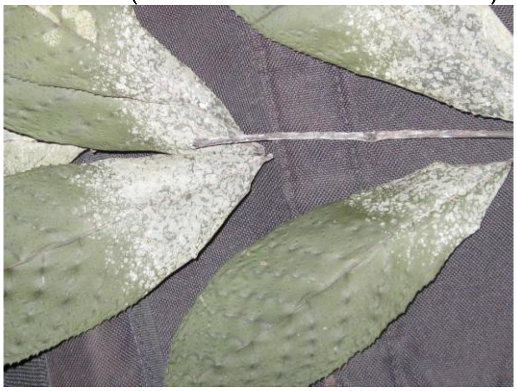

Symptoms or condition cured
Ear pain in humans
Eye problems in cattle

Part used/from which medicine is extracted
Leaves for both

General preparation method
Crush young leaves and mix with water

Method of administering medication
The concoction squeeze-dropped into the affected human ear or livestock eye

Patient age group
All age-groups

Patient gender: Both genders
