# Supplementary material for: Local indigenous knowledge about some medicinal plants in and around Kakamega forest in western Kenya
Source: F1000Res. 2012 Dec 13;1:40. Originally published 2012 Oct 31. [Version 2] doi: 10.12688/f1000research.1-40.v2 (PMC3954169; doi:10.12688/f1000research.1-40.v2)
Supplement: Medicinal plant species identified in and around Kakamega forest — Profiles of 40 putative medicinal plant species identified in and around Kakamega forest [file f1000research-1-603-s0000.tgz › Mondia_whitei.pdf]

Mondia whitei

Attributes

- Local name*:* Mukomera or Mukombela
- Family: Apocynaceae)
- Local name: White's ginger
- Plant origin: Indigenous
- Plant form: Climber

Collection site

- In relation to forest: Inside
- Forest block: Isecheno
- Specific site name: Isecheno

Collection site description

Natural (minimum-disturbance)

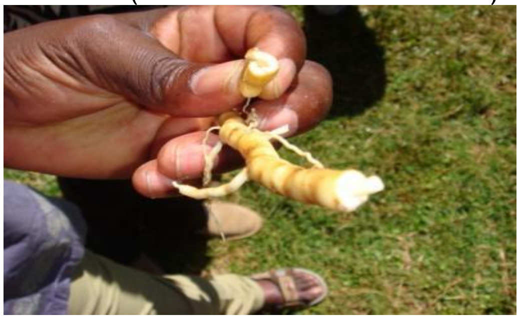

Symptoms or condition cured

- Loss of appetite
- Loss of sexual libido
- General fatigue
- Mineral deficiency

General preparation method

The roots of mature plant are dug out, cleaned and chopped up into small rods

Symptoms or condition cured

For all, indications/symptoms, the epidermal layer of the root is chewed, preferably while still fresh, as frequently as necessary

Patient age groups

All above two years of age

Patient gender: Both genders
