# Supplementary material for: Local indigenous knowledge about some medicinal plants in and around Kakamega forest in western Kenya
Source: F1000Res. 2012 Dec 13;1:40. Originally published 2012 Oct 31. [Version 2] doi: 10.12688/f1000research.1-40.v2 (PMC3954169; doi:10.12688/f1000research.1-40.v2)
Supplement: Medicinal plant species identified in and around Kakamega forest — Profiles of 40 putative medicinal plant species identified in and around Kakamega forest [file f1000research-1-603-s0000.tgz › Ocimum_kilimandscharicum.pdf]

Ocimum kilimandscharicum

Attributes

- Local name*:* Not ascertained
- Family*:* Lamiaceae
- Common name*:* Kilimanjaro basil,
- Plant origin: Indigenous
- Plant form: Shrub

Collection site

- In relation to forest: Inside
- Forest block: Buyangu
- Specific site name: Buyangu

Collection site description: Natural (minimum-disturbance) near edge

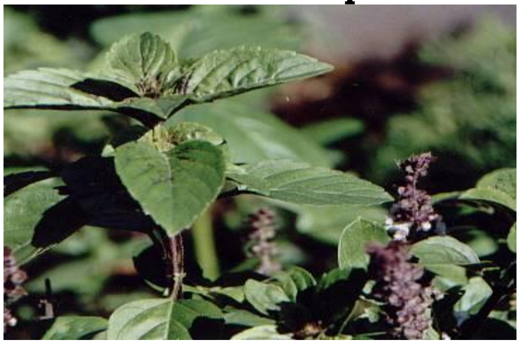

Symptoms or condition cured

Nasal congestion, colds, flu, insect bites, aches and pains

Part used/from which medicine is extracted

Leaves

General preparation method

Drying leaves and grinding to powder

Method of administering medication

- The powder is inhaled or drank with tea to relieve nasal congestion, colds and flu;
- The powder is mixed with a little cow's oil/ghee and rubbed onto insect bite wounds or massaged into painful joints

Patient age group

All age groups

Patient gender: Both genders
