# Supplementary material for: Local indigenous knowledge about some medicinal plants in and around Kakamega forest in western Kenya
Source: F1000Res. 2012 Dec 13;1:40. Originally published 2012 Oct 31. [Version 2] doi: 10.12688/f1000research.1-40.v2 (PMC3954169; doi:10.12688/f1000research.1-40.v2)
Supplement: Medicinal plant species identified in and around Kakamega forest — Profiles of 40 putative medicinal plant species identified in and around Kakamega forest [file f1000research-1-603-s0000.tgz › Olea_capensis.pdf]

Olea capensis

Attributes

- Local name: Mutukhuyu
- Common name: Elgon Olive
- Scientific name: Olea capensis
- Family: Oleaceae
- Plant origin: Indigenous
- Plant form: Tree

Collection site

- In relation to forest: Inside
- Forest block: Isecheno
- Specific site name: Shaviyoni

Collection site description: Natural (limited disturbance) area

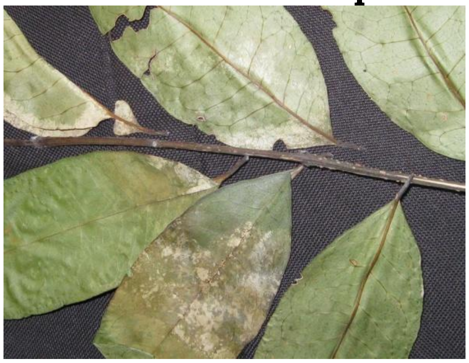

Symptoms or condition cured

Stomach-ache
Peptic ulcers

Part used/from which medicine is extracted

Bark for both ailments

General preparation method

- For stomach-ache, bark boiled in water
- For peptic ulcers, the bark is crushed and mixed with water

Method of administering medication

- For stomach-ache, the concoction drunk cold especially just before meals
- For peptic ulcers, the concoction is drunk daily till symptoms disappear

Patient age group

All age-groups

Patient gender: Both genders
