# Supplementary material for: Local indigenous knowledge about some medicinal plants in and around Kakamega forest in western Kenya
Source: F1000Res. 2012 Dec 13;1:40. Originally published 2012 Oct 31. [Version 2] doi: 10.12688/f1000research.1-40.v2 (PMC3954169; doi:10.12688/f1000research.1-40.v2)
Supplement: Medicinal plant species identified in and around Kakamega forest — Profiles of 40 putative medicinal plant species identified in and around Kakamega forest [file f1000research-1-603-s0000.tgz › Paullinia_pinnata.pdf]

Paullinia pinnata

Attributes

- Local name: Not ascertained
- Common name: Bread and cheese plant
- Family: Sapindaceae
- Plant origin: Indigenous
- Plant form: Shrub

Collection site

- In relation to forest: Inside forest
- Forest block: Buyangu
- Specific site name: Shanderema

Collection site description

Natural (undisturbed) area

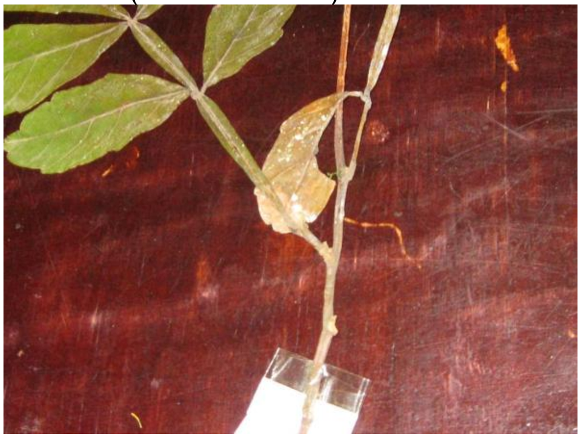

Symptoms or condition cured

Hiccup

Part used/from which medicine is extracted

Root

General preparation method

Roots crushed and mixed with water

Method of administering medication

The mixture taken orally especially when hiccups attack

Patient age group

Useful for all age groups except children under 2

Patient gender: Both genders
