# Supplementary material for: Local indigenous knowledge about some medicinal plants in and around Kakamega forest in western Kenya
Source: F1000Res. 2012 Dec 13;1:40. Originally published 2012 Oct 31. [Version 2] doi: 10.12688/f1000research.1-40.v2 (PMC3954169; doi:10.12688/f1000research.1-40.v2)
Supplement: Medicinal plant species identified in and around Kakamega forest — Profiles of 40 putative medicinal plant species identified in and around Kakamega forest [file f1000research-1-603-s0000.tgz › Paulownia_tomemtosa.pdf]

Paulownia tomemtosa

Attributes
- Local name: Musembe
- Common name: Foxglove tree/Cat's Claw
- Family: Paulowniaceae
- Plant origin: Exotic
- Plant form: Tree

Collection site
- In relation to forest: Outside
- Forest block: Ikuywa
- Specific site name: Chepsonoi

Collection site description
Farmland area

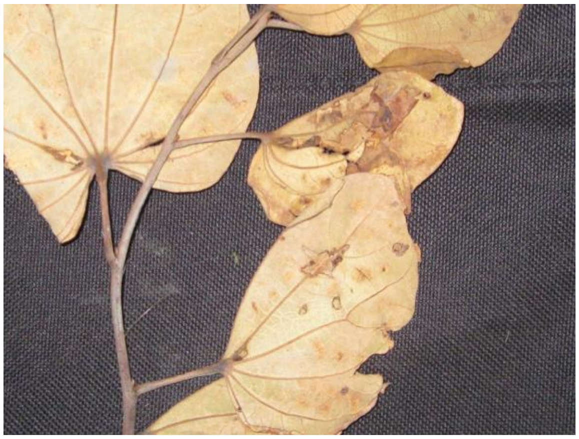

Symptoms or condition cured
Stomach problems and boils

Part used/from which medicine is extracted
Young shoots and roots

General preparation method
Roots and shoots are chopped, crushed and mixed with a little water

Method of administering medication
- The concoction is taken orally without boiling
- The leaves may also be used for fermenting porridge

Patient age group
All age groups

Patient gender: Both genders
