# Supplementary material for: Local indigenous knowledge about some medicinal plants in and around Kakamega forest in western Kenya
Source: F1000Res. 2012 Dec 13;1:40. Originally published 2012 Oct 31. [Version 2] doi: 10.12688/f1000research.1-40.v2 (PMC3954169; doi:10.12688/f1000research.1-40.v2)
Supplement: Medicinal plant species identified in and around Kakamega forest — Profiles of 40 putative medicinal plant species identified in and around Kakamega forest [file f1000research-1-603-s0000.tgz › Piper_capense.pdf]

***Piper capense***

**Attributes**
Local name: Unascertained
- Common name: Staart Pepper
- Family: Piperaceae
- Plant origin: Indigenous
- Plant form: Shrub

**Collection site**
- In relation to forest: Inside
- Forest block: Isecheno
- Specific site name: Shaviyoni

**Collection site description**
Natural (undisturbed) area

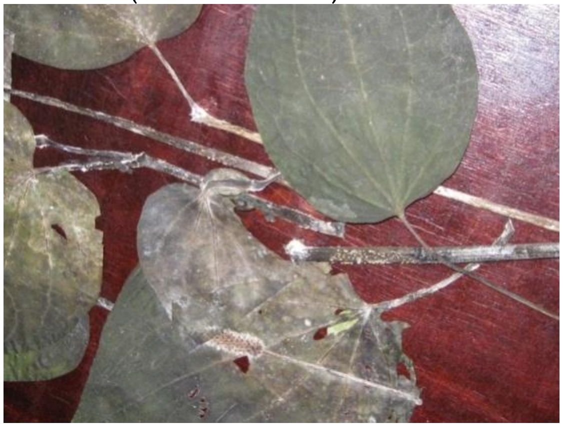

**Symptoms or condition cured**
Cough

**Part used/from which medicine is extracted**
Fruits

**General preparation method**
Whole or crushed/chopped into bits

**Method of administering medication**
- Adults simply eat/chew the fruits on experiencing attacks;
- For babies, fruit bits taken gradually administered

**Patient age group:** All age-groups

**Patient gender:** Both genders
