# Supplementary material for: Local indigenous knowledge about some medicinal plants in and around Kakamega forest in western Kenya
Source: F1000Res. 2012 Dec 13;1:40. Originally published 2012 Oct 31. [Version 2] doi: 10.12688/f1000research.1-40.v2 (PMC3954169; doi:10.12688/f1000research.1-40.v2)
Supplement: Medicinal plant species identified in and around Kakamega forest — Profiles of 40 putative medicinal plant species identified in and around Kakamega forest [file f1000research-1-603-s0000.tgz › Piper_umbellatum.pdf]

Piper umbellatum

Attributes

- Local name: Indava
- Common name: Cow-foot leaf
- Family: Piperaceae
- Plant origin: Indigenous
- Plant form: Shrub

Collection site

- In relation to forest: Inside
- Forest block: Kaimosi
- Specific site name: Shamakhokho

Collection site description
Natural (minimum-disturbance) area

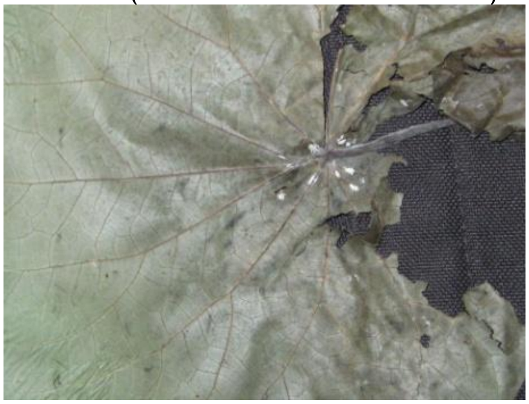

Symptoms or condition cured
Head-ache and fever

Part used/from which medicine is extracted
Leaves

General preparation method
Leaves are crushed and mixed with water

Method of administering medication
The mixture is dropped in bits into the nose

Patient age group
All age-groups older than 2 years

Patient gender: Both genders
