# Supplementary material for: Local indigenous knowledge about some medicinal plants in and around Kakamega forest in western Kenya
Source: F1000Res. 2012 Dec 13;1:40. Originally published 2012 Oct 31. [Version 2] doi: 10.12688/f1000research.1-40.v2 (PMC3954169; doi:10.12688/f1000research.1-40.v2)
Supplement: Medicinal plant species identified in and around Kakamega forest — Profiles of 40 putative medicinal plant species identified in and around Kakamega forest [file f1000research-1-603-s0000.tgz › Plectranthus_forsteri.pdf]

Plectranthus forsteri

Attributes
- Local name: Shikhokho
- Common name: Spur flower
- Family: Lamiaceae
- Plant origin: Indigenous
- Plant form: Shrub

Collection site
- In relation to forest: Outside
- Forest block: Ikuywa
- Specific site name: Ikuywa

Collection site description
Human settlement area

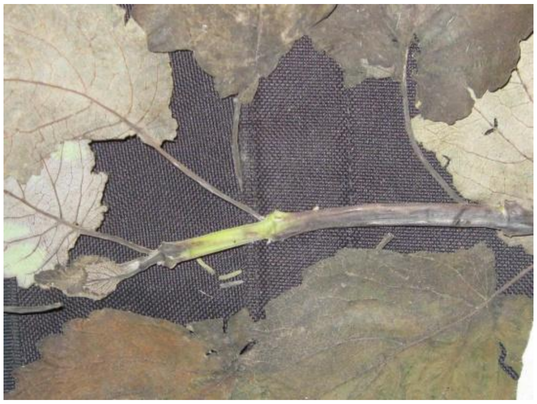

Symptoms or condition cured
Worm infection in livestock

Part used/from which medicine is extracted
Leaves

General preparation method
Leaves are crushed together with water

Method of administering medication
The mixture is administered to the affected animal orally

Patient age group
N/A

Patient gender: N/A
