# Supplementary material for: Local indigenous knowledge about some medicinal plants in and around Kakamega forest in western Kenya
Source: F1000Res. 2012 Dec 13;1:40. Originally published 2012 Oct 31. [Version 2] doi: 10.12688/f1000research.1-40.v2 (PMC3954169; doi:10.12688/f1000research.1-40.v2)
Supplement: Medicinal plant species identified in and around Kakamega forest — Profiles of 40 putative medicinal plant species identified in and around Kakamega forest [file f1000research-1-603-s0000.tgz › Prunus_africana.pdf]

Prunus africana

Attributes
- Local name: Mwiritsa
- Common name: Red Stinkwood
- Family: Rosaceae
- Plant origin: Indigenous
- Plant form: Tree

Collection site
- In relation to forest: inside
- Forest block: Buyangu
- Specific site name: Ivakale

Collection site description
Natural (undisturbed) area on the forest edge

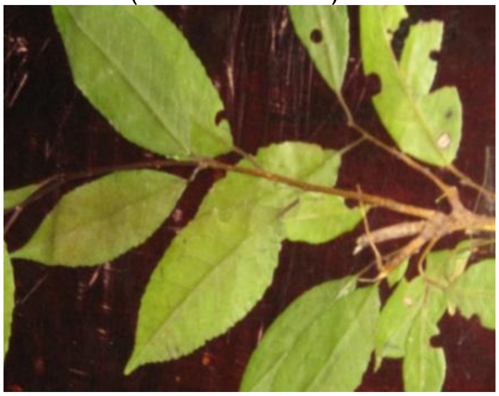

Symptoms or condition cured
- Prostrate cancer
- Stomach-ache

Part used/from which medicine is extracted
Roots and bark

General preparation method
- For prostrate cancer, simply crush bark and mix with water
- For stomach-ache, boil the mixture of crushed bark and water till extract is pinkish in colour

Method of administering medication
- For prostrate cancer, drink the mixture straight on
- For stomach-ache, allow the boiled extract to cool then drink

Patient age group

- For prostrate elderly patients
- For stomach-ache, all age groups

Patient gender
- For prostrate cancer, males only
- For stomach-ache, both genders
