# Supplementary material for: Local indigenous knowledge about some medicinal plants in and around Kakamega forest in western Kenya
Source: F1000Res. 2012 Dec 13;1:40. Originally published 2012 Oct 31. [Version 2] doi: 10.12688/f1000research.1-40.v2 (PMC3954169; doi:10.12688/f1000research.1-40.v2)
Supplement: Medicinal plant species identified in and around Kakamega forest — Profiles of 40 putative medicinal plant species identified in and around Kakamega forest [file f1000research-1-603-s0000.tgz › Rhus_natalensis.pdf]

Rhus natalensis

Attributes

- Local name: Busanguli
- Common name: Desert date
- Family: Anacardiaceae
- Plant origin: Indigenous
- Plant form: Shrub

Collection site

- In relation to forest: Inside
- Forest block: Ikuywa
- Specific site name: Ikuywa

Collection site description
Natural (minimum-impact) area

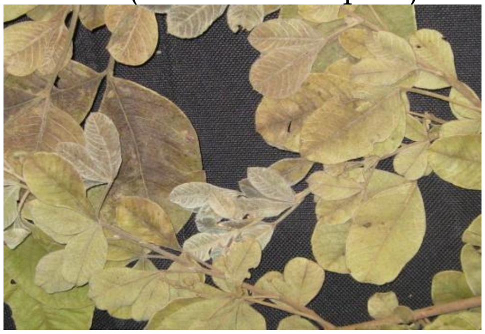

Symptoms or condition cured
Worm infections in humans and livestock

Part used/from which medicine is extracted
Roots

General preparation method
Roots are crushed together with water

Method of administering medication
The mixture is taken orally by patient, not necessarily boiled

Patient age group
N/A
Patient gender: N/A
