# Supplementary material for: Local indigenous knowledge about some medicinal plants in and around Kakamega forest in western Kenya
Source: F1000Res. 2012 Dec 13;1:40. Originally published 2012 Oct 31. [Version 2] doi: 10.12688/f1000research.1-40.v2 (PMC3954169; doi:10.12688/f1000research.1-40.v2)
Supplement: Medicinal plant species identified in and around Kakamega forest — Profiles of 40 putative medicinal plant species identified in and around Kakamega forest [file f1000research-1-603-s0000.tgz › Senecio_moorei.pdf]

Senecio moorei
Attributes
- Local name: Not ascertained
- Common name: Not ascertained
- Family: Astraceae
- Plant origin: Indigenous
- Plant form: Shrub

Collection site
- In relation to forest: Outside
- Forest block: Ikuywa
- Specific site name: Mukhumu

Collection site description
Area with some human settlement

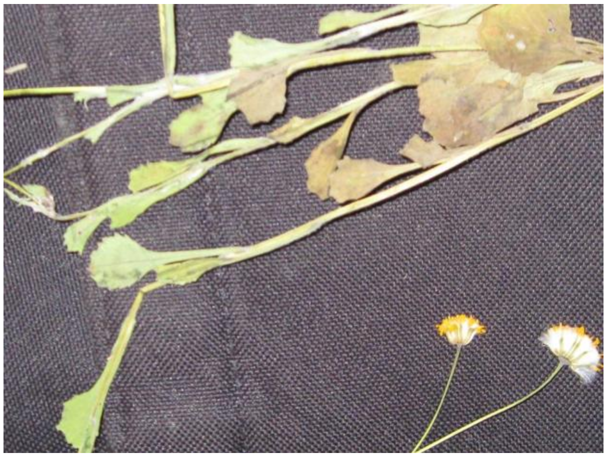

Symptoms or condition cured
Cough

Part used/from which medicine is extracted
Leaves

General preparation method
Fresh green leaves are cleaned, chopped and roasted till turns they turn into ash to which is added a pinch of salt

Method of administering medication
The patient licks up the ash at frequent intervals every day or till cough subsides

Patient age group
All age groups above 2 years

Patient gender: Both genders
