# Supplementary material for: Local indigenous knowledge about some medicinal plants in and around Kakamega forest in western Kenya
Source: F1000Res. 2012 Dec 13;1:40. Originally published 2012 Oct 31. [Version 2] doi: 10.12688/f1000research.1-40.v2 (PMC3954169; doi:10.12688/f1000research.1-40.v2)
Supplement: Medicinal plant species identified in and around Kakamega forest — Profiles of 40 putative medicinal plant species identified in and around Kakamega forest [file f1000research-1-603-s0000.tgz › Solanum_incanum.pdf]

Solanum incanum

Attributes

- Local name: Indalandalwa
- Common name: Sodom Apple
- Family: Solanaceae
- Plant origin: Indigenous
- Plant form: Shrub

Collection site

- In relation to forest: Outside
- Forest block: Ikuywa
- Specific site name: Mukhumu

Collection site description
Area with some human settlement

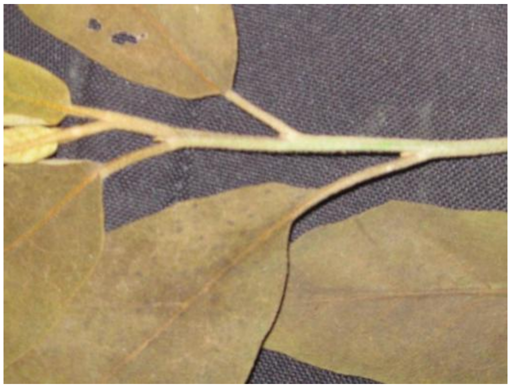

Symptoms or condition cured
Stomach-ache

Part used/from which medicine is extracted
Roots

General preparation method
Roots cleaned using water and chopped into short strands

Method of administering medication
Leaves chewed up and liquid swallowed as soon as stomach-ache strikes
Patient age group
All age groups

Patient gender: Both genders
