# Supplementary material for: Local indigenous knowledge about some medicinal plants in and around Kakamega forest in western Kenya
Source: F1000Res. 2012 Dec 13;1:40. Originally published 2012 Oct 31. [Version 2] doi: 10.12688/f1000research.1-40.v2 (PMC3954169; doi:10.12688/f1000research.1-40.v2)
Supplement: Medicinal plant species identified in and around Kakamega forest — Profiles of 40 putative medicinal plant species identified in and around Kakamega forest [file f1000research-1-603-s0000.tgz › Thunbergia_alata.pdf]

Thunbergia alata

Attributes

- Local name: Indereresia
- Common name: Black-eyed Susan vine
- Family: Acanthaceae
- Plant origin: Indigenous
- Plant form: Shrub

Collection site

- In relation to forest: Inside
- Forest block: Kaimosi
- Specific site name: Kaptik

Collection site description

Natural (minimum-disturbance) area in a glade

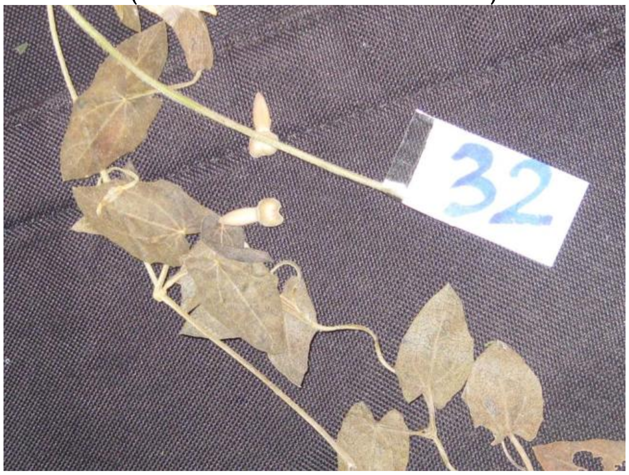

Symptoms or condition cured

Joint dislocation for both humans and livestock

Part used/from which medicine is extracted

Leaves

General preparation method

Leaves are crushed and bound into a ball then heated in open fire

Method of administering medication

Apply the ball of leaves onto the dislocated joint by pressing it firmly while still hot

Patient age group

All age groups except

Patient gender: Both genders
