# Supplementary material for: Local indigenous knowledge about some medicinal plants in and around Kakamega forest in western Kenya
Source: F1000Res. 2012 Dec 13;1:40. Originally published 2012 Oct 31. [Version 2] doi: 10.12688/f1000research.1-40.v2 (PMC3954169; doi:10.12688/f1000research.1-40.v2)
Supplement: Medicinal plant species identified in and around Kakamega forest — Profiles of 40 putative medicinal plant species identified in and around Kakamega forest [file f1000research-1-603-s0000.tgz › Toddalia_asiatica.pdf]

Toddalia asiatica

Attributes
- Local name: Unascertained
- Common name: Orange climber
- Family: Rutaceae
- Plant origin: Indigenous
- Plant form: Shrub

Collection site
- In relation to forest: Inside
- Forest block: Kaimosi
- Specific site name: Jukuye

Collection site description
Human settlement area
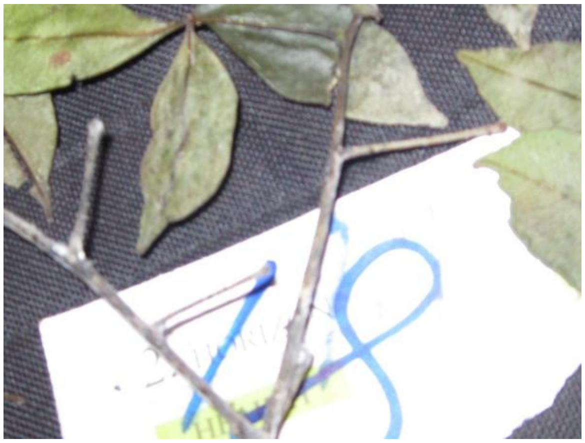

Symptoms or condition cured
Worms in cattle

Part used/from which medicine is extracted
Root

General preparation method
Roots crushed and mixed with water

Method of administering medication
Concoction administered orally to stock

Patient age group
N/A
Patient gender: N/A
