# Supplementary material for: Local indigenous knowledge about some medicinal plants in and around Kakamega forest in western Kenya
Source: F1000Res. 2012 Dec 13;1:40. Originally published 2012 Oct 31. [Version 2] doi: 10.12688/f1000research.1-40.v2 (PMC3954169; doi:10.12688/f1000research.1-40.v2)
Supplement: Medicinal plant species identified in and around Kakamega forest — Profiles of 40 putative medicinal plant species identified in and around Kakamega forest [file f1000research-1-603-s0000.tgz › Trichilia_emetica.pdf]

Trichilia emetica

Attributes
- Local name: Munyama
- Common name: Banket mahogany
- Family: Meliaceae
- Plant origin: Indigenous
- Plant form: Tree

Collection site
- In relation to forest: Inside
- Forest block: Kaimosi
- Specific site name: Shamakhokho

Collection site description
Natural (minimum-disturbance) area

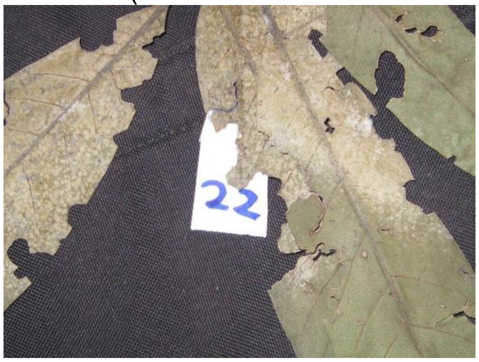

Symptoms or condition cured
Fever, stomach-ache and sexually transmitted infections
Malaria

Part used/from which medicine is extracted
Bark for all

General preparation method
Bark crushed and boiled with water till it turns pink

Method of administering medication
Mixture orally taken when cold
For malaria, half a glass of the extract is taken each morning till healed

Patient age group
All age-groups

Patient gender: Both genders
