# Supplementary material for: Local indigenous knowledge about some medicinal plants in and around Kakamega forest in western Kenya
Source: F1000Res. 2012 Dec 13;1:40. Originally published 2012 Oct 31. [Version 2] doi: 10.12688/f1000research.1-40.v2 (PMC3954169; doi:10.12688/f1000research.1-40.v2)
Supplement: Medicinal plant species identified in and around Kakamega forest — Profiles of 40 putative medicinal plant species identified in and around Kakamega forest [file f1000research-1-603-s0000.tgz › Zanthoxyllum_gilleti.pdf]

Zanthoxyllum gilleti

Attributes
- Local name: Shikhoma
- Common name: Not ascertained
- Family: Rutaceae
- Plant origin: Indigenous
- Plant form: Tree

Collection site
- In relation to forest: Inside
- Forest block: Kaimosi
- Specific site name: Shamakhokho

Collection site description
Human settlement area

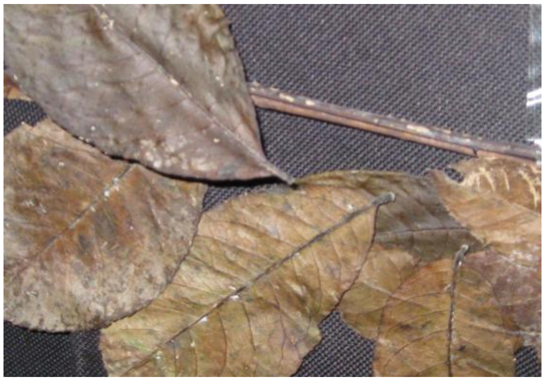

Symptoms or condition cured
Cough and chest complications associated with bacterial infections

Part used/from which medicine is extracted
Bark

General preparation method
Bark crushed and mixed with water

Method of administering medication
Mixture orally taken when cold

Patient age group
All age-groups
Patient gender: Both genders
