# Supplementary material for: Local indigenous knowledge about some medicinal plants in and around Kakamega forest in western Kenya
Source: F1000Res. 2012 Dec 13;1:40. Originally published 2012 Oct 31. [Version 2] doi: 10.12688/f1000research.1-40.v2 (PMC3954169; doi:10.12688/f1000research.1-40.v2)
Supplement: Peer review history

# Peer review: Referee response for version 2

**Published:** 2013-01-09

**Authors:** Martin Potgieter

**Affiliations:** 1 Department of Biodiversity, University of Limpopo, Sovenga, South Africa

I have reviewed the revised manuscript Local indigenous knowledge about some medicinal plants in and around Kakamega forest in western Kenya. I now find the manuscript in order, and I am satisfied that the authors have addressed all my previous reservations regarding this paper. I hereby now approve this paper.

I have read this submission. I believe that I have an appropriate level of expertise to confirm that it is of an acceptable scientific standard.

# Peer review: Referee response for version 2

**Published:** 2012-12-17

**Authors:** Hugo Asselin

**Affiliations:** 1 Sciences du développement humain et social, Université du Québec en Abitibi-Témiscamingue, Québec, Canada

I am satisfied with how the authors have modified the manuscript according to my suggestions.

A few typos remain here and there, but apart from that, I have nothing left to say. This is an interesting descriptive study that hopefully is just a first step towards a more complete and more thorough publication.

I have read this submission. I believe that I have an appropriate level of expertise to confirm that it is of an acceptable scientific standard.

# Peer review: Referee response for version 1

**Published:** 2012-11-17

**Authors:** Hugo Asselin

**Affiliations:** 1 Sciences du développement humain et social, Université du Québec en Abitibi-Témiscamingue, Québec, Canada

This is an interesting piece of work, although very incomplete. I actually hesitated between recommending acceptation or rejection, but decided to settle on the former because any new contribution on traditional knowledge related to medicinal plants should be welcomed. However, I do have several important concerns to raise about this article.

First, it should be made very clear that the study is based on the knowledge of only 3 respondents. Each of them might know a lot, but they still are just 3 respondents. Completely different results altogether could have been obtained by interviewing 3 other respondents.

Second, it is hard to evaluate the actual contribution of the study to the scientific knowledge, as no other study on traditional knowledge related to medicinal plants in Kenya or tropical Africa is cited. A quick search in Web of Science shows that 87 papers were published in the last 15 years for Kenya alone.

Third, more details should have been provided about the forests in which sampling took place. Calling them tropical is not enough. Information on species richness, for example, would have been needed to appreciate if the 40 species recorded as medicinal plants form a significant or trivial proportion of the complete species set. In addition, dominant species and forest dynamics should have been provided to facilitate comparison with other studies. Also, the choice of the forest blocks where sampling took place should have been justified.

Fourth, information should have been provided about how ethical issues were addressed. Traditional knowledge is a sensible topic (even the more so when it relates to medicinal plants) and a precautionary approach should be taken to ensure protection of intellectual property rights.

Fifth, the choice of the 3 respondents should have been explained in more details. Why only 3? Why these 3? Were they men or women? Knowledge is not shared equally between genders. Etc.

**Other comments:**

- The abstract is too general and does not provide all the relevant results.
- The English should be checked by a native speaker. Some sentences are awkward, some words are missing, and some words are uselessly repeated.
- Figure 1 does not show the effect of forest fragmentation, so the first paragraph of the “Study area” section should not imply that.
- Why wasn’t BirdLife International (2004) added to the reference list and cited properly?
- Population density should be given as a number of people per SQUARED kilometers.
- “Salaza” is not shown on Fig. 1.
- Please make clearer the distinction between “block”, “fragment”, “section”, etc.
- Herbarium voucher numbers should be provided, or, at least, the name of the herbarium where samples are kept should be given.
- A total of 40 medicinal plant species seems low. How does it compare with other studies in African tropical forests?
- Why use quotation marks when writing “cure”? This uselessly sheds a doubt on the efficacy of medicinal plants.
- I am surprised that none of the species (especially herbs) was used entirely (instead of just leaves, or fruits, or other parts).
- How were the disease classes chosen? The “vector-borne” class is not a type of disease, but rather a way of transmission. It can include digestive, psychic, or other types. Furthermore, several common ailment categories were not reported to be treated with medicinal plants. Explanations should have been provided as to why. Authors should have followed, for example, Cook’s (1995) classification : Cook FEM (1995) Economic Botany Data Collection Standard. Kew: Royal Botanic Gardens.

I have read this submission. I believe that I have an appropriate level of expertise to confirm that it is of an acceptable scientific standard, however I have significant reservations, as outlined above.

# Response

**Published:** 2012-12-14

**Authors:** Nickson Otieno

**Affiliations:** National Museums of Kenya, Kenya

Dear Dr. Asselin,

Thank you for your review. We have made the following changes in light of your comments.

**Choice of respondents:** We have now made it clear that there were 3 main focal respondents but that there were 2 other opportunistic random respondents that provided additional information for the study in each forest block, making a total of 9 respondents. Such selection was based on prior consultation with local community leaders and additional guidance by field assistants. Since this study was not meant for gauging opinions, we did not set out to interview as many respondents as possible. That is why we state that we had a key focal group of respondents chosen for their knowledge about the same, and of a minimum age that is generally recognized globally to posses the greatest of such knowledge. One of our key respondents was a practicing healer with long experience in the practice.

**Consultation of existing literature on the subject, in Kenya:** We have incorporated more references to the literature in the revised version.

**Ethical issues in data use:** Prior consent was obtained from each informant before information was obtained including information that the data would be shared widely. All respondents were duly acknowledged in the manuscript and are publicly acknowledged in the final publication. A condition for publication of the manuscript was to provide the detailed data so it was not optional not to disclose the full dataset of all the medicinal plants in detailed form.Abstract too general: We have now provided more details about the results in the abstract.Grammatical errors etc: More careful revision has been made in this regard including in-house pre-review by experienced authors.

**Figure 1 not related to fragmentation:** The reference to Figure 1 is now placed in a more explicitly relevant part of the paragraph.

**BirdLife International reference:** A more recent reference has now been included.Population density unit: This is now provided (in per square kilometers).

**Block/fragment/section: **This is now clarified as referring to forest blocks.

**Herbarium vouchers:** The plant specimens that were collected were not part of herbaria specimens and so did not have voucher numbers. They are yet to be curated and catalogued as the EA Herbarium in Nairobi is rather short of space for replicate specimens.

**Total of 40 medicinal plants:** This number of species identified has now been put in perspective by comparing with other studies elsewhere in Kenya.

**Quotation marks on “cure”:** The term “cure” has been replaced with “treatment” which we feel is more appropriate.

I am surprised that none of the species (especially herbs) was used entirely (instead of just leaves, or fruits, or other parts: In Table 1, a number of plants for which more than one part is used for treatment is provided.

**Choice of classification of disease:** The diseases were classified mainly on the basis of the parts of the body that are affected. Obviously this does not apply for “vector-borne” and “livestock” but the idea about including the former was to highlight such vector transmitted diseases, which are common in the area, such as malaria, which would otherwise easily be subsumed by the other classes since malaria presents with a multitude of symptoms. For “livestock” diseases, again this was to highlight them as non-human and compare them with the non-human ones.More details about the forest: We have now added more details in describing Kakamega forest where the study was carried out, including floral and other species status, and the overall vegetation structure.

**Selection of blocks:** We have described the rationale for the choice of forest blocks.

# Peer review: Referee response for version 1

**Published:** 2012-11-06

**Authors:** Martin Potgieter

**Affiliations:** 1 Department of Biodiversity, University of Limpopo, Sovenga, South Africa

This is potentially a very interesting article, and I think it could ultimately make a positive contribution to the evidence base. However, this paper has a number of serious flaws.

**More information and detail required:**- The abstract and results are superfluous and do not report on some of the major findings.- More can be made of the data in figure 4. Why, for example, are leaves so much used, when in the rest of Africa roots are being predominantly used?- The information from figure 3 is not reported in the results or discussion. For example it would be interesting to know why digestive was so much treated.

**Discussion, conclusion and recommendations:**- The discussion focuses most on generalities and not specifics as is found in the results.- Data on methods of preparation, administration, age and gender are not reflected in the results and discussion. This is particularly important for gender as the level of knowledge of local/rural African communities vary. Women are generally more knowledgeable in local households, but men are more knowledgeable when they are traditional healers.- References are seriously lacking in the discussion – thus no scientific authority is applied to most of the statements presented here. Thus this discussion is basically just an opinion.- Some parts of the discussion need rearranging to either the results (end of 2nd paragraph) or the conclusion (3rd paragraph).- Significant tracts of the discussion do not appear to be relevant to the study at hand, particularly the last two paragraphs of the discussion; the authors should consider removing these.- The current conclusion does not address the core data of this manuscript.- The authors should provide reasons for the points made in the ‘Recommendations’ section.

**Inconsistencies:**- Questionnaires: In the abstract and main text it is implied that multiple questionnaires are used but only one is provided.- It says in the sampling strategy that experience as a herbalist was not essential, yet in the discussion it states that indigenous medicinal knowledge is a closely guarded only passed to family members. It would have been worthwhile to know the ratio of interviewed traditional healers/practitioners vs. lay people – there is a significant difference in their level of knowledge.- In the discussion the authors state that indigenous knowledge is confined to mainly the elderly but this study targeted only people above 50. In Africa that constitutes the elderly. Thus we have no data on the knowledge level of people younger than 50. Therefore we have no data to backup this statement.

**Language:**- Some attention to the accuracy of language used is required; some words need removing, the authors should define what they mean by ‘key species’ (abstract) and appreciable knowledge (page 2) and replace the term ‘cured’ with ‘treated’.

**Notes on plant family classification:**- *Bequartiodendron oblanceolata* is from the Sapotaceae family.- Malvaceae is the correct family name for *Hibiscus spp*; Malvoideae is a subfamily.

I have read this submission. I believe that I have an appropriate level of expertise to confirm that it is of an acceptable scientific standard, however I have significant reservations, as outlined above.

# Response

**Published:** 2012-12-14

**Authors:** Nickson Otieno

**Affiliations:** National Museums of Kenya, Kenya

Dear Dr. Potgeiter, Thank you for your review. 

We have made the following changes in light of your comments.

**Title: **We have added the word “some” in the title to preclude the presumption that we sought to list all medicinal plants from Kakamega forest in the one survey.Abstract: We have restructured and re-written the abstract to reflect suggested changes. We have also clarified that we used one structured questionnaire and not many types of questionnaire.

**Introduction:** We have cited two references as suggested to support our assertion about other studies having been conducted on the subject.

**Materials and methods:** We have corrected the indicated errors and have also specified how respondents were selected, including the proportions of practitioner to lay respondents, and key to random respondents.

**Data analysis:** Suggested errors now corrected.

**Results:** *Bequartioden¬dron oblanceolata* is now assigned to the family *Sapotaceae* as has been helpfully noted by the reviewer. The reference for IUCN is now provided. The word “cure” is now replaced by “treatment”. We have also provided a clarification on methods of administering medicinal plants other than orally.

**Discussion:** We have now merged the Results with the Discussion under the new heading “Results and discussion” to make a more lucid connectivity between the two. In table 1, the Malvoidae subfamily is now corrected to *Malvaceae* family, as informed by the reviewer. The original Figure 2 depicting proportions of medicinal plant forms is now removed to avoid repeating results in text. As a result, Figure 3 becomes Figure 2 and Figure 4 becomes Figure 3. Figure 2 (new) is now reported in the text of results and discussion, together with an expoundment on the predominance of digestive-related diseases treated using the medicinal species. The new Figure3 now bears, in text, discussion as to the predominance of the use of leaves for treatments, viz-a-viz other plant parts. Table 2 is corrected as suggested; parts of the discussion suggested as not strongly related to the core data and results by reviewer, have been removed. The link between access to information on medicinal cures by local and improvement of basic healthcare is now more clearly explained. The conclusion is now more closely tied to the results of the study. Recommendations are now better justified.

**References:** Corrections on the original reference number 12 is now effected; Due to additional references (also reflected in the body text) the reference section has now been reorganized accordingly.
